# Iron–Imine Cocktail in Drug Development: A Contemporary Update

**DOI:** 10.3390/ijms25042263

**Published:** 2024-02-13

**Authors:** Judith Anane, Esther Owusu, Gildardo Rivera, Debasish Bandyopadhyay

**Affiliations:** 1 School of Integrative Biological and Chemical Sciences (SIBCS), University of Texas Rio Grande Valley, Edinburg, TX 78539, USA; judith.anane01@utrgv.edu (J.A.); esther.owusu01@utrgv.edu (E.O.); 2 Laboratorio de Biotecnología Farmacéutica, Centro de Biotecnología Genómica, Instituto Politécnico Nacional, Reynosa 88710, Mexico; gildardors@hotmail.com; 3 School of Earth, Environmental, and Marine Sciences (SEEMS), University of Texas Rio Grande Valley, Edinburg, TX 78539, USA

**Keywords:** imines, Schiff base, iron complex, anticancer, antimicrobial, antioxidant

## Abstract

Organometallic drug development is still in its early stage, but recent studies show that organometallics having iron as the central atom have the possibility of becoming good drug candidates because iron is an important micro-nutrient, and it is compatible with many biological systems, including the human body. Being an eco-friendly Lewis acid, iron can accept the lone pair of electrons from imino(sp^2^)-nitrogen, and the resultant iron–imine complexes with iron as a central atom have the possibility of interacting with several proteins and enzymes in humans. Iron–imine complexes have demonstrated significant potential with anticancer, bactericidal, fungicidal, and other medicinal activities in recent years. This article systematically discusses major synthetic methods and pharmacological potentials of iron–imine complexes having in vitro activity to significant clinical performance from 2016 to date. In a nutshell, this manuscript offers a simplistic view of iron complexes in medicinal inorganic chemistry: for instance, iron is presented as an “eco-friendly non-toxic” metal (as opposed to platinum) that will lead to non-toxic pharmaceuticals. The abundant literature on iron chelators shows that many iron complexes, particularly if redox-active in cells, can be quite cytotoxic, which can be beneficial for future targeted therapies. While we made every effort to include all the related papers, any omission is purely unintentional.

## 1. Introduction

The terms ‘imine’ and ‘Schiff base’ were invented by Albert Ladenburg and Hugo Schiff, respectively, and refer to the condensation products of carbonyl compounds (aldehydes and ketone) and amines. They are formed by the condensation of a primary amine with a carbonyl (aldehyde or ketone) compound [1] and an azomethine (-RC=N-) linkage [2]. Imines have wide applicability in many fields, especially in drug development research, because of their versatile characteristics that enable them to form a wide range of stable products. Imines can be polarized to generate an electrophilic carbon center that makes the nitrogen more nucleophilic. In mild acidic conditions, the nitrogen is protonated, making the carbon significantly electrophilic. Since their discovery by Hugo Schiff in 1864, Schiff bases have become the most important ligands in transition metal coordination chemistry due to their ease of synthesis, electronic features, solubility in many solvents, structural diversity, and abundance in biological systems [3,4,5].

The coordination of metals to organic ligands (organometallics) was not widely employed until the discovery of cisplatin and other metal-derived drugs. Many organometallic drugs have effectively been used to treat several diseases, including cancer, diabetes, and ulcers, and in imaging studies, etc. Metal–Schiff base complexes have improved antimicrobial, antioxidant, anti-inflammatory, antibacterial, and anticancer activity relative to their free Schiff base ligands [6,7]. Schiff base ligands coordinate with metal ions and stabilize them in various oxidation states. Depending on their dipole moment, solubility, enzymatic action, and cell permeability, they can increase biological activity [5]. The challenge, however, is that some of these therapeutics have raised concerns due to the fatal side effects they confer on patients. The need for new, less toxic, and more potent organometallic drugs has led to extensive research on iron–imine complex formation. So far, iron–imine complexes have been found to exhibit effective biological activities [8]. For instance, Sarkar et al. found a significant photocytotoxicity of an iron(III)–Schiff base complex (obtained from thiosemicarbazide and vitamin B6) against cervical cancer cells (HeLa) through the intracellular generation of ROS [9]. Also, some iron(III)–Schiff base complexes derived from aminophenol/aminobenzene and salicylaldehyde have shown better antibacterial and antifungal activity when compared to antibacterial and antifungal standard drugs, chloramphenicol and terbinafine, respectively [10].

It is worth mentioning that iron is the fourth most abundant (5.6%) element in the Earth’s crust [11]. It is important for the normal functioning of mammalian cells because iron plays essential roles in many dynamic biological processes that occur in the human body, like DNA synthesis, metabolism, respiration, electron transport, and erythropoiesis, among others [12,13], making their participation in mammalian cells vital for appropriate cellular function [14,15]. It is, therefore, a safer alternative for developing organometallic drugs. This review outlines the syntheses and pharmacological potential of iron–imine complexes.

## 2. Bioactivity of Imine–Iron Complexes

Imine–iron complexes provide an intriguing insight into the future of organometallic chemistry. Limited attention was given to organometallic drugs until recently when some metal-containing drugs were discovered to be useful in the battle against various diseases like cancer, antimicrobial resistance diseases, oxidative stress [3], HIV [16], bacterial (malaria), fungal, and viral infections [17], tuberculosis [18], diabetes, rheumatoid arthritis, and cardiovascular diseases [19]. Among the exciting tapestry of organometallic compounds, imine–iron complexes stand out with their bright threads of fascination and ability. These unique compounds attach iron atoms to ligands with the intriguing imine functional group, which is a nitrogen–carbon double bond generated by an amine and a carbonyl compound. Imine–iron complexes are adaptable building blocks with high promise in catalysis, biomimicry, magnetic materials, and beyond. This distinguishing trait endows them with an enticing combination of properties, making them the focus of significant research and interest in the sector.

### 2.1. Imine–Iron Complexes as Anticancer Agents

The success of cisplatin as a potent anticancer drug led to researchers exploring and discovering platinum-based drugs like carboplatin and oxaliplatin. The downside of these platinum-based drugs is their adverse side effects and drug resistance. This shifted attention to other metal-based anticancer drugs, particularly iron-based complexes, after the prolific activity of naturally occurring iron–bleomycin and ferrocenium salts like trichloroacetate and ferrocenium picrate was discovered. Their effectiveness was attributed to the oxidative DNA damage they caused by upsetting the oxidative homeostasis in cancer cells [11]. Iron–imine complexes provide a unique and potential avenue for cancer therapy due to their tailored delivery and different mechanisms of action, which include altering iron metabolism, producing reactive oxygen species (ROS), and blocking key enzymes. They additionally enhanced tumor penetration and retention and have the ability to overcome drug resistance.

Iron–imine complexes and other organometallic complexes have been found to have potent anticancer activity [20].

**Scheme 1:**
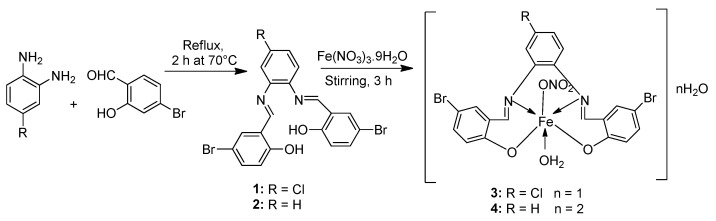
Synthesis of imine–iron complexes **3** and **4** with anticancer activity.

El-Lateef et al. synthesized two tetradentate dibasic chelating imine–iron complexes (**3** and **4**, Scheme 1) from the reaction of **1** and **2** with Fe^3+^ salt. The free ligand (**1** and **2**) and its synthesized complexes (**3** and **4**) were investigated for their in vitro cytotoxic effect against MCF-7, HepG-2, and HCT-116 cancer cell lines at different concentrations. These tested compounds had activity on breast carcinoma cells, with the cytotoxicity of the complexes being higher than that of their free ligands. Compound **4** showed the highest cytotoxicity activity against MCF-7, HepG-2, and HCT-116 (5.14, 6.75, and 4.45 µM, respectively), comparable to the standard drug doxorubicin, which had the activity of 4.10, 5.15, and 4.35 µM, respectively, and could be used as a tumor drug candidate (Table 1). The cytotoxicity of metal complexes is assumed to be due to their ability to bind DNA, hence disrupting its structure, causing replication and transcription processes to be inhibited, and eventually damaging the cancer cells (Table 1) [21].

**Scheme 2:**
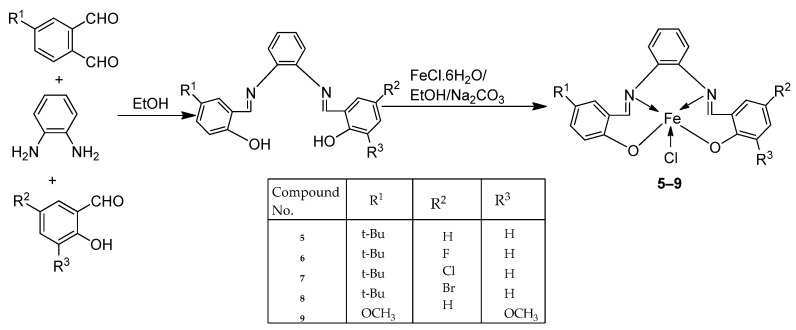
Synthesis of unsymmetrical tetradentate imine–Fe(III) complexes **5**–**9**.

Nguyen et al. [22] synthesized unsymmetrical tetradentate imine–Fe(III) complexes (**5**–**9**, Scheme 2) by coordinating the imine ligands with FeCl_3_·6H_2_O and tested them on KB and Hep-G2 human cancer cell lines. The iron–imine complexes showed excellent cytotoxicity for KB and Hep-G2 (IC_50_ < 20 µM). The presence of substituted groups in the salicyl rings affects the electrical properties and bulk of the complexes. Complex **5**, which did not have the substituted group in the second salicyl ring, exhibited the best cytotoxic activity for KB and Hep-G2 (0.68 and 0.83 µM, respectively), even better than the standard compound ellipticine, which showed an activity of 1.14 and 2.11 µM, respectively (Table 1) [22].

**Figure 1:**
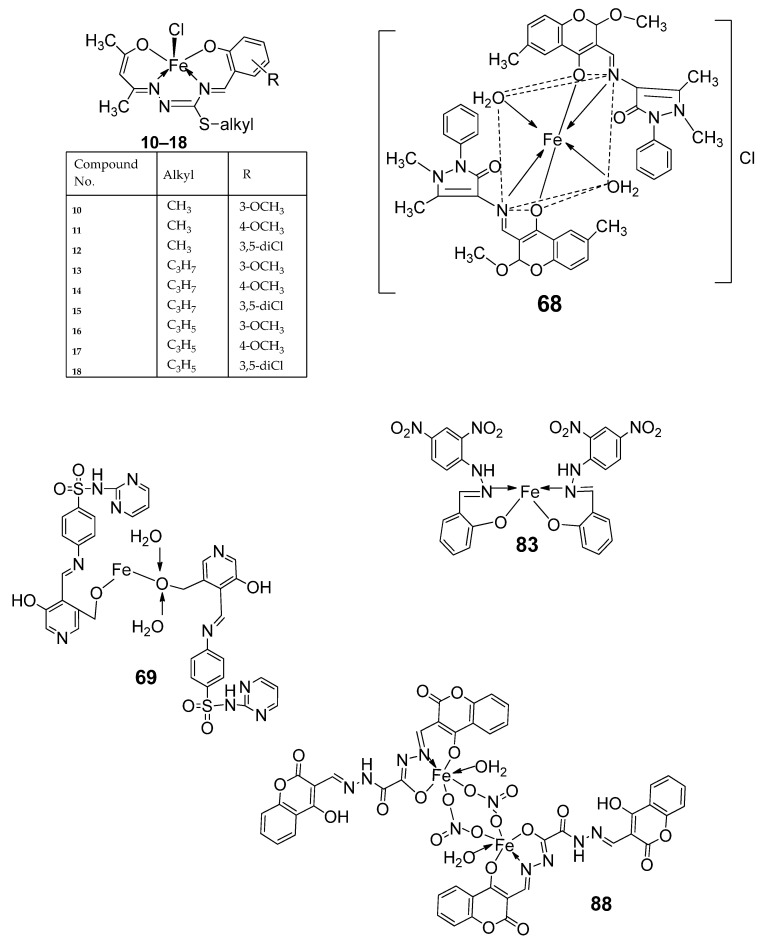

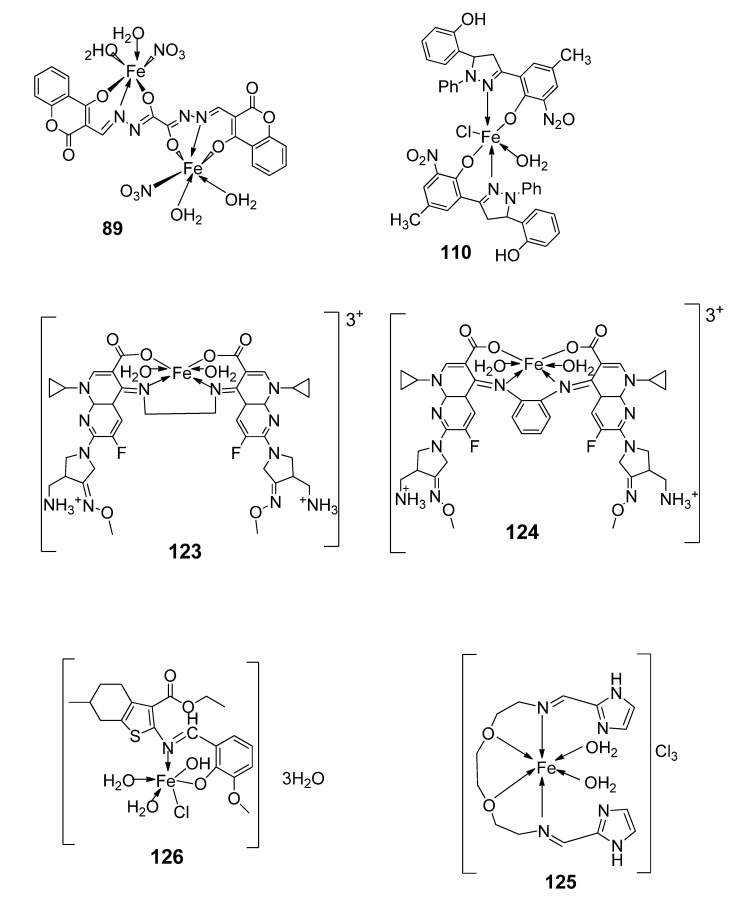
Some medicinally privileged iron–imine complexes.

Nine iron(III) complexes (**10**–**18**, Figure 1) were synthesized by Kalındemirtaş et al. The in vitro cytotoxicity activity of the iron complexes was investigated on P3HR1, K562, JURKAT, HUVEC, and 3T3 cell lines. The complexes **11**, **14**, **16**, and **17** showed a better cytotoxicity effect (in the range of 4.81–14.05 μM) on the K562 cell line than the standard imatinib, which had an activity of 9.67 µM. Five complexes had significantly lower IC50 values than the positive control (imatinib) for P3HR1 cells (Table 1). Complexes **12**, **15**, and **18**, which had a 3,5-dichloro substituent, could not compete with imatinib. All the synthesized complexes were ineffective against the JURKAT cell line in the studied concentrations. Different cells may die in different ways, and cancer cells of different types might respond very differently to the same treatment. P3HR1 and JURKAT are lymphoid cells with T- and B-lymphocytes of origin, respectively, whereas K562 is myeloid. T-cell lineage-derived leukemia includes a diverse range of neoplasms. They are typically more aggressive than their B-cell counterpart, differing in clinicopathological characteristics and biological function, and are marked by resistance to conventional chemotherapy and a bad prognosis for the patients [23]. Studies have also shown miRNAs to be critical regulators in tumorigenesis [24,25]. When exposed to chemotherapeutic drugs that are commonly used in T-cell leukemia/lymphoma treatment, like cisplatin, cytarabine, doxorubicin, and cyclophosphamide, JURKAT cells’ expression of miR181a increased along with AKT activation [26]. The different results obtained in the JURKAT cells may be due to these differences [27].

**Scheme 3:**
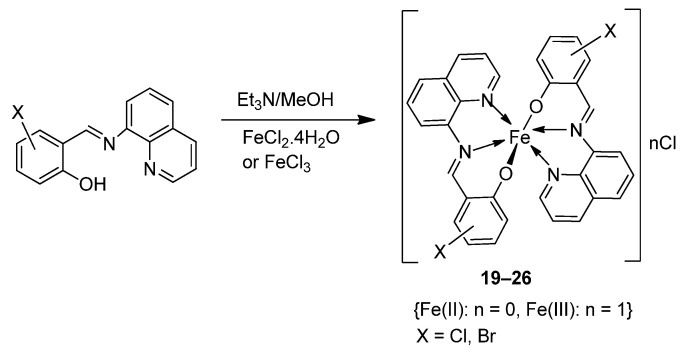
Synthesis of Fe(II) and Fe(III) complexes **19**–**26** with antitumor activity.

Wongsuwan et al. synthesized a series of Fe(II) complexes (**19**–**22**, Scheme 3) and Fe(III) complexes (**23**–**26**, Scheme 3) by coordinating imine derived from 8-aminoquinoline and salicylaldehyde with Fe(II)/(III) chloride (Scheme 3). Imine complexes were screened against the A549 human lung adenocarcinoma cell line. The imine ligand showed no anticancer activity, but the complexes showed moderate-to-high anticancer activity against A549 cells with IC_50_ values ranging from 10 to 34 µM. Complex **22** showed the highest antiproliferative activity of 10 µM, which is higher than that of two well-known commercial drugs, etoposide (19 µM) and cisplatin (16 µM) (Table 1). Transition metal complexes can bind to DNA through both covalent and non-covalent interactions. Complex **6** showed very high DNA affinity and induced high levels of ROS (hydroxyl and peroxyl radicals) in A549 cancer cells. These two factors together contributed to the antiproliferative activity of complex **6**. Therefore, DNA binding and intracellular ROS that cause macromolecular or DNA damage and cell death are potential mechanisms by which the complexes enter A549 cells [28].

**Scheme 4:**
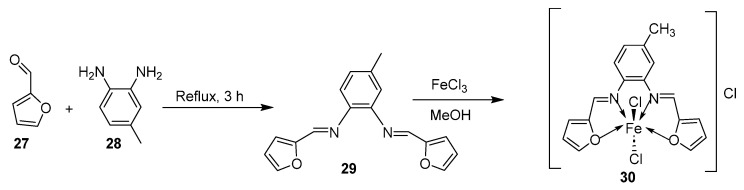
Synthesis of iron(III)–imine complex **30**.

An iron(III) complex (**30**, Scheme 4) of novel imine ligand **29** was synthesized by Ismail et al., and its cytotoxicity activity against the Hep-G2 cell line was evaluated. The Fe(III) complex (**30**, Scheme 4) showed an enhanced antitumor activity (7.31 μg/mL) compared to that of the solo Schiff base (IC_50_ = 27 μg/mL). Still, compared to the standard drug vinblastine, which showed a value of 2.93 μg/mL, its antitumor activity was moderate (Table 1) [29].

**Scheme 5:**
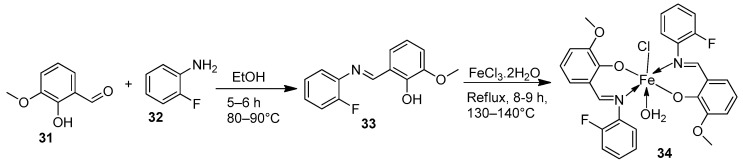
Synthesis of iron complex **34**.

The ligand (**33**) and its metal complex (**34**, Scheme 5) were synthesized by Kavitha et al. and studied over three cancer cell lines: human pancreatic carcinoma (MiaPaCa-2), human cervical adenocarcinoma (HeLa), and murine melanoma cancer cells (B16F10), and one normal cell N1H/3T3 (fibroblast cells). The IC_50_ value for the complex, 106.26 μg/mL, was beyond 100 µg/mL, signifying very low anticancer activity against the selected cancer cell lines (Table 1). DNA binding studies showed that the complex had a low binding affinity for the DNA, which could have been responsible for its low antitumor potency [30].

**Scheme 6:**
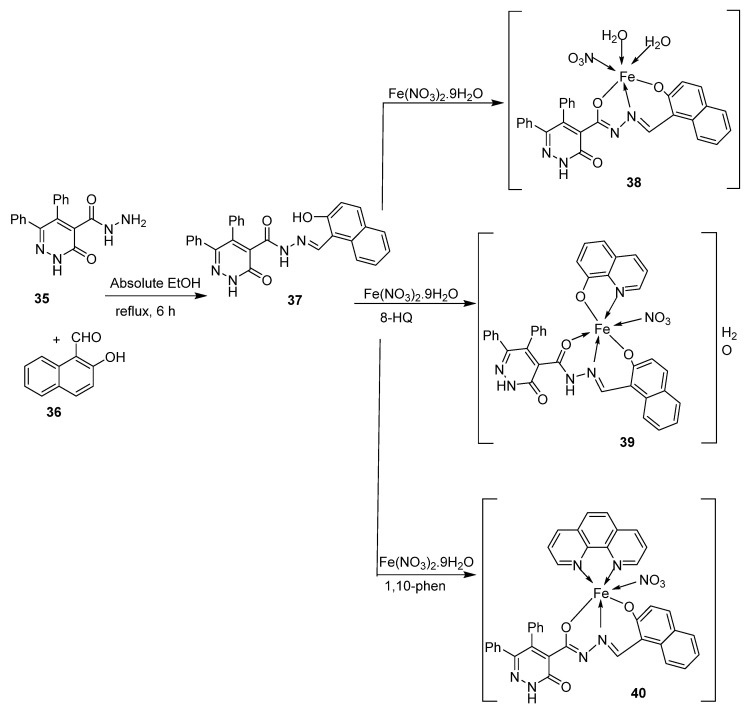
Synthesis of iron complexes **38**–**40**.

Abdelrahman et al. synthesized new nano-Fe(III) complexes (**38**–**40**) of pyridazinone-acid hydrazone ligand **37** and new mixed-ligand complexes using 8-hydroxyquinoline or 1, 10-phenanthroline (Scheme 5) as an auxiliary ligand. The complexes and the imine ligand were tested against hepatocellular carcinoma cell lines (HepG-2 cells) for their antitumor activity in vitro. The imine ligand showed strong antitumor activity against the HepG-2 cells, but the activity of the synthesized iron complexes (**38**–**40**) was insignificant (Scheme 6, Table 1). Complex **37** showed an antitumor activity of 3.80 μg/mL against HepG-2, whilst the standard drug, Cisplatin, showed an activity of 3.27 μg/mL (Table 1) [31].

**Scheme 7:**
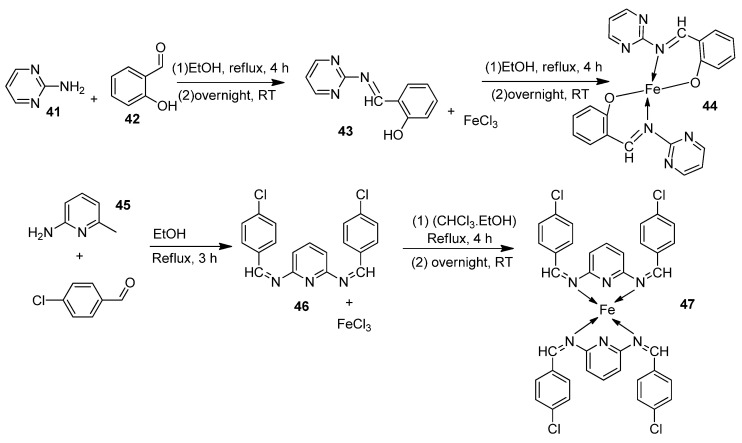
Synthesis of complexes **44** and **47**.

Farhan et al. synthesized two heterocyclic imine ligands (**43** and **46**) and prepared complexes (**44** and **47**, Scheme 7) from the fusion of the imine ligands with Fe(III), resulting in an octahedral geometry and paramagnetic complex (**44** and **47**). The ligands and imine complexes were investigated for their anticancer potency against the L20B cell line at a 4000 μg/mL concentration. The iron complex **44** demonstrated a high anticancer activity of 8.7 μg/mL against the (L_20_B) cell line. The anticancer activity of **47** was comparatively low, 22.9μg/mL (Table 1). The results were not compared with a standard anticancer agent [32].

**Table 1:** Product, synthesis conditions, and in vitro anticancer activity (IC_50_ in µM) of selected iron–imine complexes compared to the respective positive controls ^†^.

Entry No.	Complex No.	Structures	Synthesis Condition	Complex and Positive Control	Cancer Cell Lines			Ref.
1.	**3**, **4**	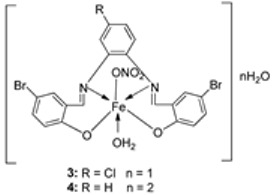	EtOH Reflux, 2 h Stirring		MCF-7	HepG-2	HCT-116	[21]
				**3**	21.35 ± 0.12	27.70 ± 0.11	15.75 ± 0.07	
				**4**	5.14 ± 0.05	6.75 ± 0.12	4.45 ± 0.14	
				Doxorubicin	4.10 ± 0.13	5.15 ± 0.07	4.35 ± 0.15	
2.	**5**–**9**	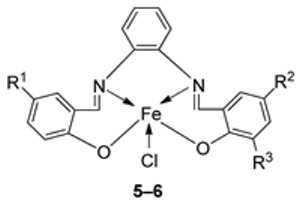	EtOH Reflux, 3 h		KB	HepG-2		[22]
				**5**	0.68 ± 0.05	0.83 ± 0.05		
				**6**	3.25 ± 0.16	7.05 ± 0.25		
				**7**	1.84 ± 0.10	6.07 ± 0.22		
				**8**	2.76 ± 0.17	19.78 ± 1.07		
				**9**	1.95 ± 0.13	2.38 ± 0.17		
				Ellipticine	1.14 ± 0.06	2.11 ± 0.12		
3.	**10**–**18**	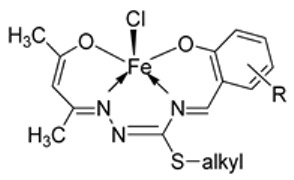	Stirring, 30 min		K562	P3HR1	JURKAT	[27]
				**10**	>25	>25	>25	
				**11**	9.25 ± 0.42	5.61 ± 0.19	>25	
				**12**	22.24 ± 0.06	8.09 ± 0.62	>25	
				**13**	>25	>25	>25	
				**14**	4.81 ± 0.15	11.98 ± 0.69	22.79 ± 0.54	
				**15**	>25	22.4 ± 0.47	>25	
				**16**	14.05 ± 0.31	5.72 ± 0.28	>25	
				**17**	5.04 ± 0.18	11.47 ± 0.42	22.0 ± 0.39	
				**18**	>25	21.03 ± 0.39	>25	
				Imatinib	9.67 ± 0.49	23.74 ± 1.02	3.73 ± 0.21	
4.	**19**–**26**	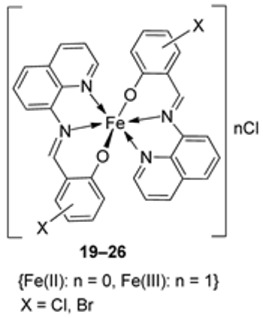	0 °C, 7 days		A549			[28]
				**19**	30 ± 1.1			
				**20**	30 ± 7.7			
				**21**	28 ± 2.0			
				**22**	28 ± 2.0			
				**23**	28 ± 2.0			
				**24**	10 ± 2.1			
				**25**	34 ± 4.7			
				**26**	32 ± 1.5			
				Etoposide	19 ± 1.3			
				Cisplatin	16 ± 1.9			
5.	**30**	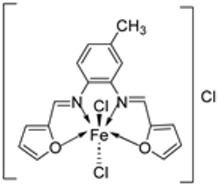	Reflux, 3 h Stirring, 2 h		Hep-G2			[29]
				**30**	7.31			
				Vinblastine	2.93			
6.	**34**	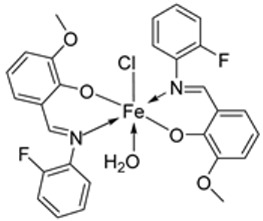	Reflux, 8–9 h		Hela	MiaPaCa-2	B16F10	[30]
				**34**	106.26 ± 0.5	112.13 ± 0.6	104.15 ± 1.2	
7.	**38**–**40**	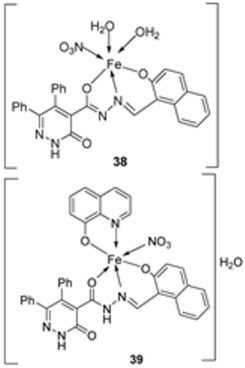	Stir, 2 h Reflux, 12–15 h		Hep-G2			[31]
				**37**	3.8058.00			
				**38**	R			
				**39**	R			
				**40**	3.27			
				Cisplatin				
8.		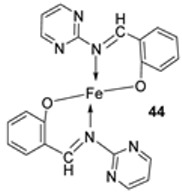	Reflux, 3–4 h		L_20_B			[32]
	**44**			**44**	8.70			
	**45**			**45**	13.20			
	**46**			**46**	18.4			
	**47**			**47**	22.9			

^†^ C_50_ values written as these have been reported in the literature.

### 2.2. Imine–Iron Complexes as Antimicrobial Agents

Antimicrobial agents are compounds that can inhibit (stop or reduce) the growth of microorganisms such as bacteria, fungi, protozoa, etc. Microbial resistance to antibiotics and other antimicrobial drugs has become one of the major health concerns globally. Due to their distinct characteristics and action methods, research has focused on imine–iron complexes as promising agents [33]. Imine–iron complexes appear as possible game changers in the fight against microorganisms, outperforming many traditional antimicrobials. They are considered possible game changers because of the following:

1. Novel mechanism of action: Traditional antibiotics typically target specific bacterial functions like cell wall synthesis or protein translation, which can lead to resistance development as bacteria mutate those targets, imine–iron complexes employ diverse mechanisms, including iron starvation, DNA cleavage, and reactive oxygen species (ROS) generation, making it harder for bacteria to develop resistance.
2. Broad-spectrum activity: Traditional antibiotics often have specific targets, limiting their effectiveness against different types of bacteria, whilst imine–iron complexes can exhibit activity against a wider range of bacteria, including multi-drug-resistant strains, due to their multiple attacking mechanisms.
3. Biofilm disruption: Traditional antibiotics may struggle to penetrate bacterial biofilms, protective communities are resistant to many drugs, whilst imine–iron complexes show potential to disrupt biofilms, exposing bacteria within to attack further.
4. Reduced side effects: Traditional antibiotics can harm beneficial gut bacteria and other healthy cells due to their broad targeting, whilst imine–iron complexes can be designed to be more selective for bacterial targets, potentially reducing the side effects on human cells.

In recent years, metal complex-based antibiotic compounds have become a promising avenue in drug development. According to research, 21% of the metal compounds examined exhibited antibacterial action against typical strains of *Candida* and *Cryptococcus* strains [34]. Therefore, there is an urgent need to develop next-generation antimicrobial agents, and imine-iron complexes can be the right avenue to move forward because these complexes are known for their antimicrobial activity. The observed microbial activity can also be traced to (i) the concept of cell permeability and the chelation process which reduces the polarity of a metal ion; (ii) the chelation process which increases electron delocalization on the chelate ring and enhances the lipophilicity of the complex, granting it easy penetration through microbial cells; (iii) the toxicity of metal ions [35]; (iv) the introduction of an azomethine linkage improves the hydrophobicity and liposolubility of the molecules; and additional factors that contribute to the improved biological activity are the solubility, conductivity, and dipole moment of the metal ion [36,37,38]. The antimicrobial activities of Schiff bases and their metal complexes have been studied against different bacterial and fungal strains [39].

**Scheme 8:**
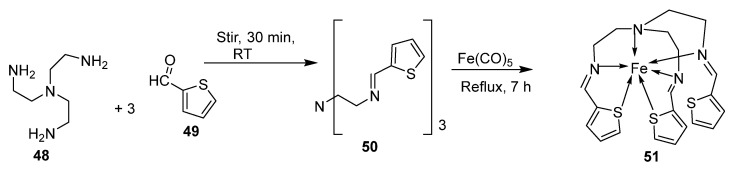
Synthesis of complex **51**.

Rahmatabadi et al. synthesized the iron metal complex (**51**, Scheme 8) of imine ligand (**50**), prepared by condensing **48** with **49**. Imine–iron complex (**51**) was tested for its in vitro antibacterial potency against Gram-negative *Escherichia coli* (*E. coli*) and *Pseudomonas aeruginosa* (*P. aeruginosa*) bacteria and Gram-positive bacteria *Bacillus cereus* (*B. cereus*) and *Staphylococcus aureus* (*S. aureus*) with tetracycline, gentamicin, chloramphenicol, and cephradine as a standard control. Complex **51** showed enhanced activity compared to the free ligand (**50**). It had the highest antibacterial activity against *B. cereus* (29 mm) and *S. aureus* (14 mm), which was higher than the activity of the standard drug tetracycline against *B. cereus* (11 mm) and *S. aureus* (9 mm), but it showed moderate activity against *E. coli* (14 mm) and *P. aeruginosa* (14 mm), which was for both bacteria (Table 2). These recorded activities of the complexes are due to the more pronounced lipophilic nature of the metal centers in the complexes [40,41].

**Scheme 9:**
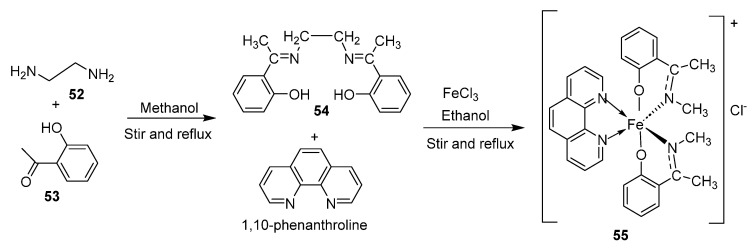
Synthesis of complex **55**.

Shukla et al. synthesized imine ligand **54** by condensing **52** with **53** in a 1:2 molar ratio. Imine ligand **54**, 1,10-phenanthroline, and FeCl_3_ were combined to form a mixed-ligand iron complex (**55**, Scheme 9) and analyzed for their antibacterial activity against Gram-negative bacteria *E. coli* in comparison to amoxicillin and chloramphenicol standard drugs. Complex **55** exhibited enhanced activity against *E. coli* (29 mm) compared to **54** (23 mm). Still, it showed moderate antibacterial activity compared with standards chloramphenicol and amoxicillin, which showed inhibition zones of 39 mm and 41 mm, respectively (Table 2). The action of metal ions on the normal cell membrane may cause the metal complex’s increased activity. Either the microbes’ cells’ impermeability or variations in the ribosomes of microbial cells determine the complex’s ability to combat *E. coli*. The outcome could be explained by considering the chelation theory, which suggests that chelation could facilitate a complex’s capacity to pass across a cell membrane [42,43].

El-Lateef et al. explored **3** and **4** (Scheme 1) for their antibacterial potency against three selected bacterial strains: *S. marcescence*, *E. coli*, and *M. Luteus*. Both complexes showed high antibacterial activity against the selected bacteria, with **4** showing the highest antibacterial activity against *M. luteus* (2.50 μg/mL) (Table 2). The values of the activity of standard drugs were not provided. The activity of the complexes was high compared to that of the free ligands (**1** and **2**) due to the chelation theory. The polarity of the metal ion is greatly reduced during chelation due to electron delocalization throughout the entire chelate ring system and partial sharing of its positive charge with the hetero-donor atoms of the ligand [44,45]. The different activities displayed by the complexes against the various microbes are due to the differences in the chemical makeup of the microorganisms’ cell walls [21].

**Scheme 10:**
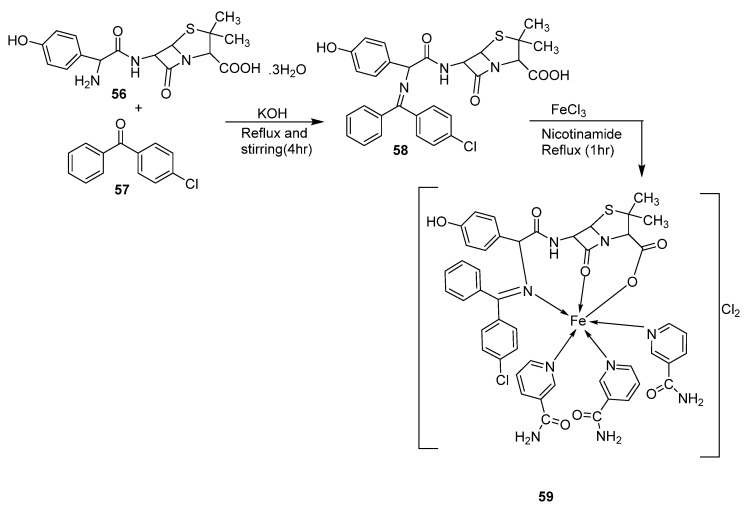
Synthesis of complex **59**.

The iron complex **59** (Scheme 10) was synthesized by Karem et al., and its antibacterial potency was evaluated against *P. aeruginosa*, *E. coli*, *S. aureus*, and *B. subtilis*. The iron complex showed no activity for all the bacterial strains except for *E. coli*, against which it showed an activity of 25 μg/mL. This value was higher than that of the free ligand, which showed an inhibition of 2.5 μg/mL (Table 2). The observed increase in activity against *E. coli* can be explained by Tweedy’s theory [46]. The results obtained were not compared to a standard drug [47].

**Scheme 11:**
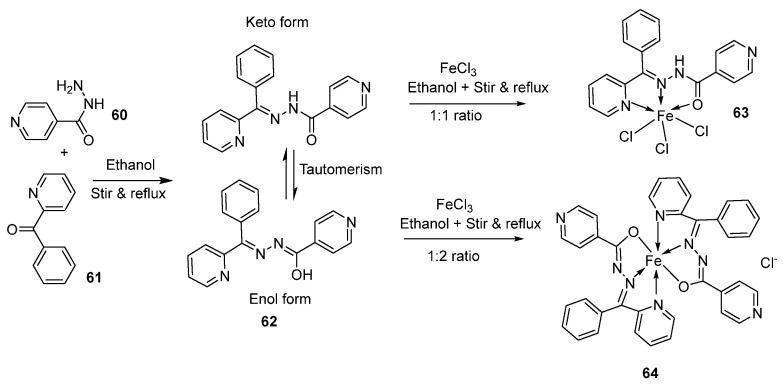
Synthesis of complexes **63** and **64**.

The imine ligand **62** synthesized by the condensation of **60** and **61** (Scheme 11) was complexed with Fe(III) by Shukla et al. to form two imine–iron complexes, **63** and **64**. The complexes were tested against Gram-positive bacteria, *B. subtilis*, and Gram-negative bacteria, *E. coli*, with amoxicillin as a standard. The complex **64** (14 mm and 18 mm, respectively) showed enhanced activity compared to the free ligand (11 mm and 15 mm, respectively) against *B. subtilis* and *E. coli*, and this activity of **64** was similar to that of the standard amoxicillin (16 mm and 20 mm, respectively) against the same microbes. The antimicrobial activity of complex **63** is similar to that of ligand **62** against the selected microbes (Table 2). The result shows that chelation makes it easier for these complexes to traverse the cell membrane, which is consistent with Tweedy’s chelation theory. Due to the partial sharing of the metal ion’s positive charge with donor groups during chelation, the metal ion’s polarity will be lowered, and the delocalization of π-electrons over the entire chelate ring will be increased. This improves the complex’s lipophilicity, favoring its passage through the lipid membrane, and interferes with the metal binding sites in the microbes’ enzymes [48].

**Scheme 12:**
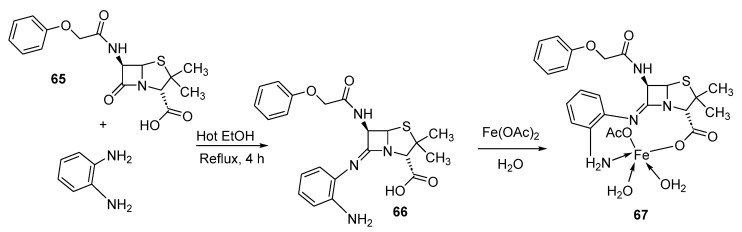
Synthesis of iron complex **67**.

The imine ligand **66**, prepared by the condensation of **65** with 1,2-diaminobenzene and its iron complex (**67**, Scheme 12), was synthesized by Anacona et al. and analyzed for its antibacterial activity against pathogenic bacteria Gram-positive *Enterococcus faecalis* (*E. faecalis*) ATCC 29212 and *S. aureus* ATCC 25923 and clinical isolates of *Streptococcus viridans* (*S. viridans*), *Enterococcus* Sp., and methicillin-resistant *S. aureus* (MRSA). The iron complex (**67**) showed enhanced activity against all the selected microbes compared to the ligand **66**. It exhibited very good antibacterial activity against methicillin-resistant *S. aureus* (15 mm), whereas the standard drug and free ligand showed no activity at all. The complex under study [49] showed moderate activity against the other bacterial strains (Table 2). The moderate-to-high activity of the complex is attributed to not only the chelation theory but also other factors like the nature of the metal ion, the type and quantity of donor atoms, stereochemistry, chelate stability, and pharmacokinetic factors [50].

Pahontu et al. synthesized an Fe(III) complex (**68**, Figure 1) and tested its antimicrobial activity against Gram-positive bacteria *S. aureus*, *B. cereus*, *and E. faecalis* and Gram-negative bacteria *E. coli* and *A. baumannii*, as well as fungal strains *Candida albicans* (*C. albicans*), *Candida krusei* (*C. krusei*), and *Cryptococcus neoformans* (*C. neoformans*). The MIC values of the iron–Schiff base complex obtained correlated with very low antibacterial activity against all the bacterial strains selected compared to the standards used (furacilin, ciprofloxacin, and amikacin). The complex showed improved antifungal activity against *C. albicans* and *C. neoformans*, with values of 0.0156 and 0.0078μg/mL, respectively, compared to the standard drugs nystatin (*C. albicans* = 0.032 μg/mL, *C. neoformans* = 0.032 μg/mL) and miconazole (*C. albicans* = 0.016 μg/mL, *C. neoformans* = 0.0162 μg/mL) used in studies (Table 2) [6]. The lack of activity of the synthesized complex against the bacterial strain is unclear. Still, its impressive antifungal activity against *C. albicans* can be attributed to the metal ion’s ability to reduce binding energy while increasing the binding affinity of the microbe protein, hence interrupting its biological processes [10].

Mumtaz et al. complexed iron(II) with an imine ligand to form the iron(II) metal complex **69** (Figure 1), which was investigated for its antimicrobial activity against *E. coli*, *Enterobacter aerogenes* (*E. aerogenes*), *S. aureus*, *B. pumilus*, *K. oxytoca*, and *C. butyrium*. The iron complex’s zone of inhibition of the various bacterial strains was quite small, demonstrating low antibacterial activity towards the bacteria. Still, these values were higher than those of the free imine ligand. Complex **69** showed an activity of 12, 10, and 9 (mm) against *E. coli*, *E. aerogenes*, and *C. barium*, respectively, and the ligand showed an activity of 14, 12, and 12 (mm), respectively (Table 2). The complex’s enhanced activity compared to the ligand can be explained by chelation theory. [46,51].

**Scheme 13:**
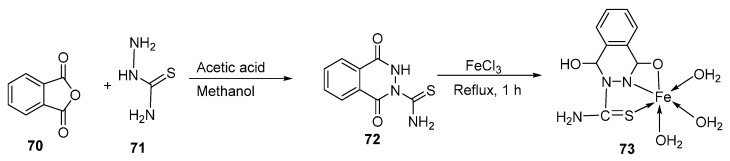
Synthesis of complex **73**.

Al-Wasidi et al. synthesized an iron–Schiff base complex by complexing imine ligand **72** with Fe(III) to form an octahedral iron complex (**73**, Scheme 13) which was investigated for its antibacterial and antifungal activity against Gram-positive *B. subtilis*, *S. pneumonia*, and *S. aureus*, Gram-negative *E. coli* Sp. and *Pseudomonas* Sp., and fungal strains *Aspergillus niger* (*A. niger*) and *Penicillium* Sp. The iron–imine complex **73** showed enhanced antibacterial activity relative to the free ligand **72** with a great zone of inhibition against *S. pneumonia* (7–10 mm) and *S. aureus* (7–10 mm). It demonstrated low inhibition against the selected fungal strains (Table 2). The results obtained were not compared to any standard drug [52].

**Scheme 14:**
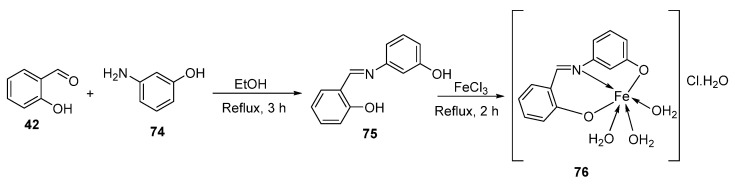
Synthesis of imine–iron complex **76**.

El-Sonbati et al. synthesized imine–iron complex **76** with ligand **75** (Scheme 14) and evaluated its antimicrobial activity against Gram-positive bacteria *B. subtilis* and *S. aureus*; Gram-negative *bacteria* such as *Salmonella* sp, *P. aeruginosa*, and *E. coli*; and fungal strains *A. fumigatus* and *C. albicans*. For all the bacterial strains selected, complex **76** showed similar antibacterial activity to the free imine ligand and low antibacterial activity when compared with the selected standard drugs ampicillin and gentamycin (Table 2). Against the fungal strains *C. albicans* and *A. fumigatus*, the complex showed improved inhibition of 16 mm and 18 mm, respectively, compared to the free imine ligand (13 mm and 15 mm, respectively). This inhibition was low compared to the standard antifungal drug amphotericin, which had an activity of 25 mm and 23 mm zones of inhibition, respectively. The improved antifungal activity of complex **76** in relation to the free ligand (**75**) can be explained by the chelation theory, where the chelation of the ligand causes an increase in the lipophilicity properties of the metal chelate, enhancing its ability to permeate the lipoid layers of the microbe membrane blocking the metal binding site [4,46].

**Scheme 15:**
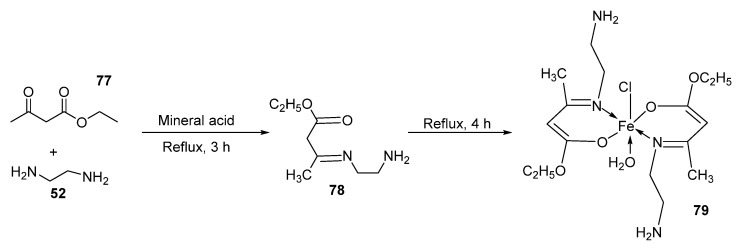
Synthesis of imine–iron complex **79**.

Kumar et al. synthesized the imine–iron complex **79** (Scheme 15) of imine ligand **78** and evaluated its antibacterial activity against Gram-positive *P. aeruginosa* and Gram-negative *S. aureus* bacteria. The complex (**79**) showed an improved antibacterial activity compared to the free ligand (**78**) against *S. aureus* and *P. aeruginosa* with a zone of inhibition of 14 mm and 11 mm, respectively, whereas the ligand showed an inhibition of 8 mm and 6 mm, respectively. Complex **79** had a comparable zone of inhibition to that of the standard drugs ampicillin against *S. aureus* (14 mm) and chloramphenicol against *P. aeruginosa* (8 mm) (Table 2) and can be further investigated as an antibacterial drug candidate. The improved antibacterial activity of the complex can be attributed to the chelation theory [46,53].

**Scheme 16:**
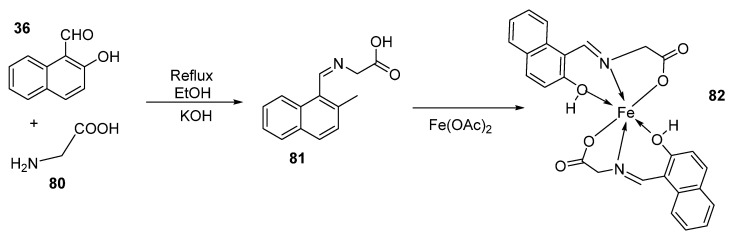
Synthesis of iron complex **82**.

Fe(II) complex **82** (Scheme 16) of compound **81** was synthesized by Shinde et al., and upon investigating its antimicrobial activity against Gram-positive bacteria *S. aureus* (ATCC 29737), Gram-negative bacteria *E. coli* (ATCC 25922), and fungal strains *C. albicans* (MTCC 277) and *A. niger* (MCIM 545), it was found to possess high activity against *E. coli* (ATCC25922) and *S. aureus* with an MIC value of 10 μg/mL against both bacteria. This value is the same for the standard drug gentamicin, which also showed an activity of 10 μg/mL. Complex **82** also showed improved activity against both fungal strains *C. albicans* (MTCC 277) and *A. niger* (MCIM 545) with an MIC value of 10 μg/mL when compared with the standard drug fluconazole, which showed an activity of 20 μg/mL against both strains (Table 2) [2]. The reason for the exceptional antimicrobial potency of the synthesized complex (**82**) was not stated, but it could be due to chelation theory [46] and the good binding interaction of **82** with the proteins of the selected strains.

Mukhtar et al. synthesized an imine–iron metal complex (**83**, Figure 1), and its antimicrobial activity was investigated against five bacterial isolates, *E. coli*, *S. aureus*, *P. aureginosa*, *K. Pneumoniae*, and *S. aureus*, and three fungal species, *F. solani*, *A. fumigate*, and *C. albicans.* The results of these studies revealed that the complex showed the highest antibacterial activity against *E. coli* (14 mm) at a concentration of 1000 μg/mL but moderate activity against the other bacterial isolates (Table 2). Its antifungal activity was quite low. It inhibited the growth of *C. albicans* and *F. solani* by 7 mm at a concentration of 2000 μg/mL and 12 mm at a concentration of 4000 μg/mL, respectively. It showed no antifungal activity against *A. fumigate* at the studied concentrations. The ligand showed no zone of inhibition against *E. coli* and *P. aeruginosa* at the given concentrations. It, however, showed similar activity to the complex against *S. aureus* (12 mm) at a concentration of 1000 μg/mL. It also showed no activity against all the selected fungal strains (Table 2). The results obtained in this study were not compared to any standard drug [54]. The reason for the improved antimicrobial activity of the synthesized complex can be attributed to the chelation theory [46].

**Scheme 17:**
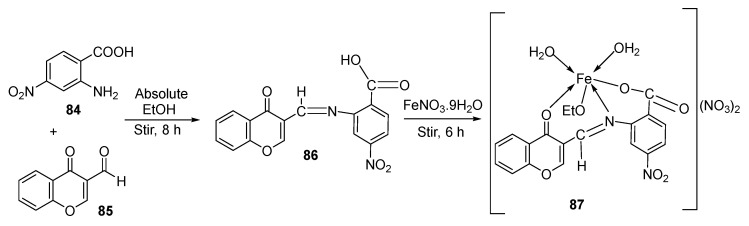
Synthesis of complex **87**.

The synthesis of a chromone imine nano-complex of Fe(III) (**87**, Scheme 17) was conducted by Shebl et al., and its antimicrobial activity was tested against microorganisms such as *E. coli*, *P. vulgaris*, *K. pneumonia*, *S. aureus*, and *C. albicans*. The results showed that the iron complex (**87**) has moderate activity against fungal species *C. albicans* (8 μg/mL) when compared to free ligand **86** (4 μg/mL) and a standard (2 μg/mL); it, however, exhibited very low activity (>50 µg/mL) toward all the selected bacterial stains in comparison to the standard drug doximycin which showed activity in the range of 2–4 μg/mL (Table 2) [3].

Knittl et al. synthesized two different iron–imine complexes (**88** and **89**, Figure 1) and evaluated them for their antimicrobial activity against Gram-positive bacteria *S. aureus* (ATCC25923), Gram-negative *P. phaseolicol* (S97), and fungal species *F. oxysporium* using cephalothin, chloramphenicol, and cycloheximide, respectively, as standard antibiotics. The results indicate that **88** exhibits higher antibacterial and antifungal activity against the selected microbes, *S. aureus* (37 mm), *P. phaseolicol* (26 mm), and *F. oxysporium* (31 mm), in comparison to **89**, which showed an inhibition of 32 mm, 23 mm, and 30 mm against *S. aureus*, *P. phaseolicol*, and *F. oxysporium*, respectively. These values suggest moderate antibacterial and antifungal activities of the complexes compared to the standard antibiotic and antifungal drugs cephalothin, chloramphenicol, and cycloheximide. Both synthesized complexes showed improved antimicrobial activity against the selected microbes compared to the free ligand (Table 2). Chelation tends to increase the ligand’s effectiveness as a potent antibacterial agent. From the results obtained, there is evidence for the relationship between the structure of the complexes and their activity. Antimicrobial activity is enhanced by binuclear complexes rather than acyclic complexes, revealing that these complexes are biologically more efficient and, therefore, can be useful as new drugs. It is also discussed that the chemical geometry of compounds is important in explaining the biological activity of the complexes [55].

**Scheme 18:**
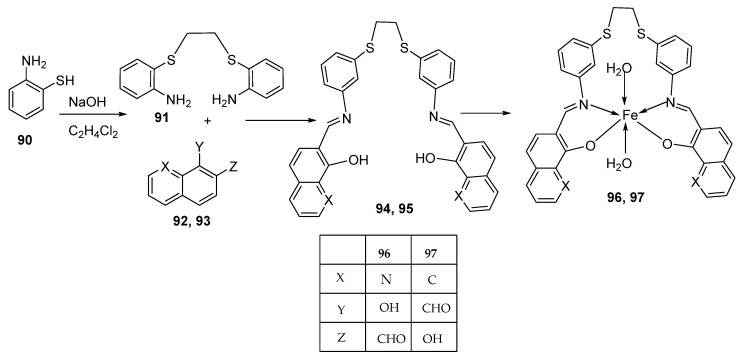
Synthesis of complexes **96** and **97**.

Alosaimi et al. synthesized two symmetrical imine ligands (**94** and **95**) and reacted each with FeCl_3_·6H_2_O to form mononuclear octahedral Fe(III) complexes **96** and **97** (Scheme 18). The complexes were screened for their antibacterial activity against Gram-positive bacterial strains *S. epidermidis*, *S. aureus*, and *E. faecalis* and Gram-negative bacterial strains *P. aeruginosa*, *E. coli*, and *P. mirabilis*. Antifungal activity was also determined against the common pathogenic fungal strain *C. albicans*. The tested Schiff base ligands (**94** and **95**) exhibited negligible antibacterial action against Gram-positive bacterial species with growth-limiting diameters of 15 mm. They also showed no antifungal activity against *C. albicans*. Iron complex **96** showed higher antibacterial activity against the Gram-positive bacterial strain *S. epidermidis* (14 mm) than iron complex **97** (with a zone of inhibition of 12 mm). The Gram-negative bacterial strain *P. mirabilis* was slightly inhibited by both iron complexes, **96** (8 mm) and **97** (22 mm), but all the other strains were resistant to both complexes. The complexes exhibited low antibacterial activity compared to the standard antibiotic agent, amoxicillin. The antibiotic agent inhibited *S. epidermidis* and *P. mirabilis* with zones of inhibition of 28 mm and 44 mm, respectively. The fungus *C. albicans* was resistant to both iron complexes and showed no significant antifungal activity (Table 2). Overtone’s permeability concept and Tweedy’s chelation theory can both be used to explain why coordination compounds have more activity than their parent ligands [46,56]. The complexes become more permeable when a metal ion is present because they dissolve in lipids and enter the cell more readily, causing negative changes in the cell environment and its enzymes, further hindering the microbe’s growth. Additionally, the metal complexes impede the production of proteins by impeding the cell’s respiration process, further inhibiting the organism’s growth. Additionally, the probability of hydrogen bonds forming between the azomethine linkage and the cell components will negatively impact the cell’s normal functions [57,58].

**Scheme 19:**
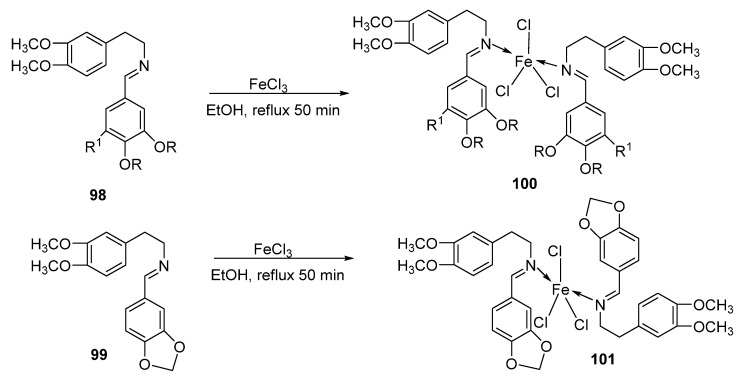
Synthesis of complexes **100** and **101**.

Iron(III) was complexed with two imine ligands (**98** and **99**, Scheme 19) by Naureen et al. to form iron complexes **100** and **101**. The ligands and their complexes were evaluated for their antibacterial activity against Gram-positive *P. aeruginosa* and Gram-negative *E. coli* and *S. aureus* using tetracycline as the standard drug. Their antifungal activity was also evaluated against *C. albicans* and *C. glabrata* with nystatin as the standard drug. The antimicrobial activity of the synthesized complexes was enhanced when compared to their free Schiff ligands. Both complexes showed similar inhibition against all the bacterial strains used in this research, but **101** showed better activity against *S. aureus* (20 mm) and *C. albicans* (24 mm) compared to **100**, which showed a zone of inhibition of 16 mm and 20 mm, respectively. The complexes showed low antibacterial activity when compared with the standard drug tetracycline. Both complexes showed higher antifungal activity against *C. albicans* than the standard drug nystatin (19 mm) and could be investigated as promising antifungal drug candidates (Table 2) [59]. The chelation theory can explain the improved activity of the complexes compared to their free Schiff ligands [46].

**Scheme 20:**
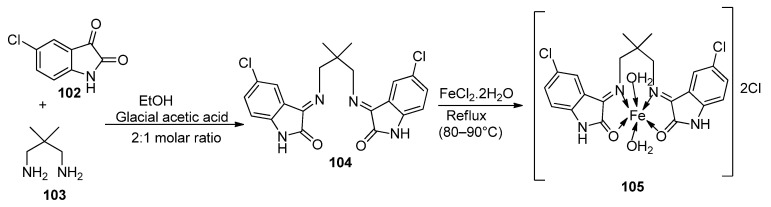
Synthesis of complex **105**.

Singh et al. synthesized an imine ligand by the condensation of compounds **102** and **103** in the molar ratio of 2:1, respectively. The synthesized ligand (**104**) was complexed with iron to form an octahedral **105** (Scheme 20) and tested against *S. epidermidis*, *E. coli*, *A. flavus*, *A. niger*, and *C. lunata* to validate its antibacterial and antifungal potentials. The complex showed better antibacterial activity against the selected bacterial and fungal strains than the Schiff base ligand. The complex showed the highest activity against *A. niger* (16 mm) and low activity against *E. coli* fungal strains (15 mm) (Table 2) [60]. The improved activity of the complexes in relation to their ligands can be explained based on Overtone’s concept and Tweedy’s chelation theory [46,56].

Kavitha et al. evaluated **34** (Scheme 5) for its antibacterial and antifungal activity against Gram-positive *Staphylococcus* Sp. and *Bacillus* Sp. as well as Gram-negative *E. coli* and *Pseudomonas* bacterial strains and fungal strains *Macrophamina phaseolina* (*M. phaseolina*) and *Sclerotium rolfsii* (*S. rolfsii*). The iron complex **34** showed enhanced biological activity against the bacterial and fungal strains, *B. subtilis* (4 mm), *E. coli* (4 mm), and *M. phaseolina* (14 mm) compared to the ligand (**33**), which showed an activity of 1, 1, and 8 mm, respectively. However, these activities of the complex are low when compared to the standard antibiotic streptomycin and the standard antifungal agent mancozeb (Table 2). The concept of Overtone explains the increased activity of complexes [51].

**Scheme 21:**
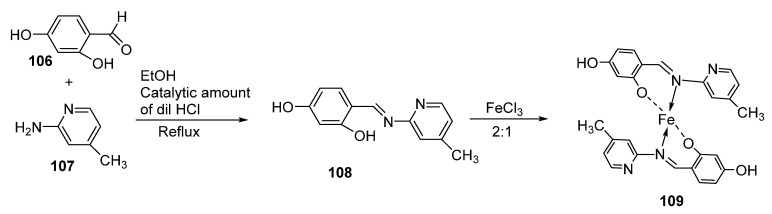
Synthesis of complex **109**.

Borase et al. synthesized a pyridine imine transition metal complex of Fe(III) (**109**, Scheme 21) by reacting metal salts (FeCl_3_) with compound **108**. The complex was evaluated for its antibacterial and antifungal potency against Gram-positive bacteria *S. aureus* and Gram-negative bacteria *E. coli*, as well as three fungal strains, *C. albicans*, *A. niger*, and *F. moniliforme.* The iron complex (**109**) showed potent antifungal activity against *A. niger* (15.80 mm) when compared to the standard amphotericin-B (15.78 mm). Complex **109** showed low antifungal and antibacterial activity against *C. albicans* (7.44 mm) and *S. aureus* (3.02 mm). The complex was resisted by *E. coli* and *F. moniliforme* (Table 2). The antimicrobial activities of the ligand were not provided in this study, so a comparison could not be made [61].

Deshmukh et al. reported an imine ligand and used it to synthesize the Fe(III) complex **110** (Figure 1) and analyzed its antimicrobial activity against Gram-positive *S. aureus* and *S. pyrogenes* and Gram-negative *E. coli* and *S. typhi* pathogens. The complex showed the highest activity against *S. aureus* (22 mm) and the least activity against *E. coli* (16 mm) (Table 2). The results of antibacterial activity were not compared with ligands or standard drugs, and hence a comparison could not be made [62].

**Scheme 22:**
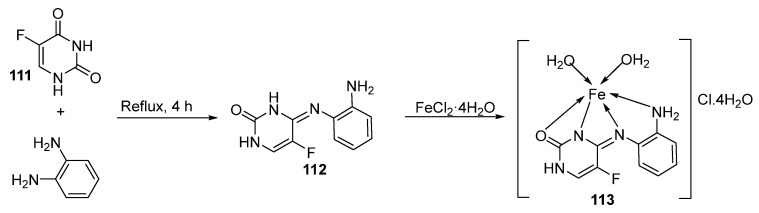
Synthesis of complex **113**.

Savcı et al. synthesized a Schiff base ligand (**112**) and complexed it with FeCl_2_⋅4H_2_O to form the transition metal imine–iron complex **113** (Scheme 22). Compounds **111**, **112**, and **113** were evaluated for their antimicrobial activity against Gram-positive *B. subtilis*, *S. aureus*, and *B. megaterium* and Gram-negative *E. aerogenes*, *E. coli*, *P. aeroginosa*, and *K. pneumonia* bacterial strains and fungal strains *C. albicans*, *Y. lipolytica*, and *S. cerevisiae.* The results indicate that both **111** and **112** show better antibacterial activity against *B. subtilis* ATCC 6633 (zone of inhibition of 40 mm and 30 mm, respectively) than the synthesized iron complex **113** (21 mm) at a concentration of 0.2 mg/mL. The complex showed antibacterial activity against *E. aerogenes* (30 mm) and *P. aeruginosa* (36 mm) only at an elevated concentration of 1 mg/mL but did not show significant antifungal activity against the selected fungal strains (Table 2). Only **111** showed activities against *K. pneumonia*(36 mm) at a 0.2 mg/mL concentration. Compounds **111** and **112** were found to have superior antibacterial activity compared to all the standard antibiotic drugs against *B. subtilis*, *B. megaterium*, *E. aerogenes*, and *P. aeruginosa*. The sizes and load distributions of the metal ions, the shape of the metal chelate, and the potential for redox, as well as the increased lipophobicity of the molecules, may all affect the impact of the metal complexes on microbes [63]. However, it does not appear to be possible to simply attribute the bactericidal activity to the metal complex structure [64,65].

**Scheme 23:**
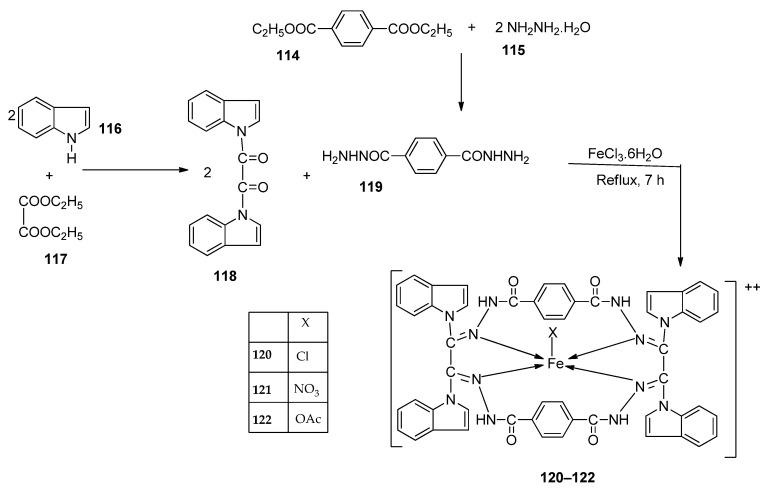
Synthesis of complexes **120**–**122**.

Kumar et al. synthesized an imine ligand and complexed it with FeCl_3_·6H_2_O, Fe(NO_3_)_3_·9H_2_O, and Fe(OAc)_3_·2H_2_O to form iron complexes **120**, **121**, and **122** (Scheme 23), respectively. The synthesized complexes were screened for their antimicrobial activity against *S. aureus* and *B. subtilis* (as Gram-positive bacteria), *P. aeruginosa*, *E. coli*, and *Salmonella typhi* (as Gram-negative bacteria), and fungi *Rizoctonia sp.*, *Aspergillus sp.*, and *Penicillium* Sp. Complex **122** demonstrated the highest antibacterial activity against *S. aureus* (62 mm) and *P. aeruginosa* (65 mm). Complex **120** showed the highest activity against *E. coli* (41 mm) and *S. typhi* (42 mm). The antibacterial activity of complexes **121** and **122** was higher against the Gram-positive bacteria than against the Gram-negative bacteria (Table 2), and this is due to the difference in the structure of the cell walls. Gram-negative cells have more complex cell walls than Gram-positive ones (Table 2). The results for antifungal screening show that **122** has high antifungal potency against *Aspergillus sp* (80 mm) and *Penicillium* Sp. (66 mm), even better than that of the standard drug miconazole with an inhibition of 57 mm and 65 mm, respectively, at a concentration of 1.0 mg/mL. Complexes **120** and **121** showed moderate antifungal activity toward the selected strains (Table 2). Generally, the ligand demonstrated moderate activity and the complexes displayed moderate-to-high activity toward all the organisms compared to standard drugs. This could be due to the presence of the -NH group, which is believed to impart the biological system’s transformation reaction and plays a significant role in biological activity. Chelation theory also explains the enhanced activity of the complexes compared to the ligand [46,66].

Mohamed et al. synthesized the novel octahedral iron–imine complex **123** (Figure 1) and evaluated its antimicrobial potential on the bacterial strains *Clavibacter michiganensis*, *Xanthomonas campestris*, and *Bacillus megaterium* and fungal strains *Monilinia fructicola*, *Penicillium digitatum*, and *Colletotrichum acutatum*. The free Schiff base ligand showed better antibacterial activity against all the selected bacterial strains than its iron complex. The ligand exhibited higher antibacterial activity against *C. michiganensis* (32 mm) than the standard drug tetracycline (30 mm). It also showed similar activity to tetracycline against *B. megaterium* and *X. campestris* (Table 2). Also, complex **123** (Figure 1) showed enhanced antifungal activity against *M. fructicola*, (62.5 mm) and *P. digitatum* (62.5 mm) compared to both the free Schiff base ligand (36.0 and 28.0 mm, respectively) and the standard antifungal agent azoxystrobin (45.3 and 58.1 mm, respectively) and can be considered as an antifungal drug candidate. The microbicide impact of the investigated compounds may result from the chemical structure of the free ligand as well as the toxicity of the investigated metal ions [67,68]. The increased antimicrobial activity of freshly synthesized metal chelates was explained by the principle of cell permeability of the microbes [35].

The imine–iron complex **124** (Figure 1) was synthesized by Elshafie et al., and its biological activity was evaluated against both human and phytopathogens. Antimicrobial analysis was conducted on pathogenic bacterial strains *E. coli*, *B. cereus*, *Pseudomonas fluorescens*, and *P. aeruginosa* and phytopathogenic fungi *Monilinia fructicola*, *Aspergillus flavus*, *Penicillium italicum*, and *Botrytis cinerea.* The antibacterial activity of **124** was dose-dependent. It showed the highest antibacterial activity against *B. cereus* with a measured zone of inhibition of 14 mm at a concentration of 100 µg/mL, higher than that of both the ligand (12 mm) and tetracycline (12 mm). Complex **124** inhibited the growth of *P. aeruginosa* (8 mm) and *P. fluorescens* (12 mm) only at a higher concentration of 200 µg/mL. Generally, the free imine showed better antibacterial activity than the metal complex **124**. Complex **124** exhibited no antifungal activity against *M. fructicola.* Still, it showed enhanced activity against *B. cinerea* (6.7 mm) at a concentration of 400 µg/mL, whereas at the same concentration, it was resisted by the free ligand. The activity of both the Schiff base ligand and the complex was low when compared to the standard natural antifungal drug cycloheximide (Table 2). The acquired antimicrobial test findings demonstrated that the tested ligands and their metal complexes have the capacity to suppress the growth of all strains under study in a dose-dependent manner. In particular, the chemical structure of the free ligand itself and the toxicity of the metal ions under study could both contribute to the fungicidal effects of the compounds under study [63,64]. Chelation theory can also explain the enhanced activity of the complex. Also, the investigated gemifloxacin ligand and its metal complexes’ capacity to block the DNA gyrase and DNA topoisomerase IV enzymes may potentially be related to their antifungal and antibacterial action [69,70].

Ismail et al. synthesized an imine–iron complex (**30**, Scheme 4) and evaluated it for its in vitro antibacterial activity against Gram-positive bacteria *S. aureus* and *B. subtilis*, Gram-negative bacteria *P. vulgaris* and *E. coli*, and fungi *A. flavus and C. albicans.* The imine ligand exhibited better antibacterial and antifungal activity against all the microbial strains studied than **30**. Complex **30** showed lower antibacterial and antifungal activity against *S. aureus*, *E. coli*, and *C. albicans* with a zone of inhibition of 17, 19, and 15 mm, respectively, compared to the selected antibacterial standard drugs gentamycin (*S. aureus* = 24 mm and *E. coli* = 30 mm) and ketoconazole (*C. albicans* = 20 mm). The ligand had high action against *C. albicans*, displaying antimicrobial activity (25 mm) superior to that of the ketoconazole standard (20 mm). Additionally, the ligand’s inhibition zone value against *B. subtilis* is 25 mm, which is comparable to the standard gentamycin (26 mm) (Table 2). The reason for the reduced antimicrobial efficiency of the complex was not stated [29].

Abdelrahman et al. evaluated complexes **38**, **39**, and **40** (Scheme 6) for their antimicrobial activity against Gram-positive bacteria *S. aureus* and *B. subtilis*, Gram-negative *S. typhimurium* and *E. coli* bacteria, and unicellular *C. albicans* and multicellular *A. fumigatus* fungi. The free ligand was ineffective against all the studied microbes except for *C. albicans*, which had an inhibition zone of 8 mm. Iron complexes **38** and **40** showed no activity against the selected bacterial strains. Complexes **38** and **39** showed moderate antifungal activity against *C. albicans* with an inhibition zone of 14 mm and 22 mm, respectively; these values were high when compared to the free ligand, which showed an inhibition of 8 mm. The lipophilicity of compounds significantly influences the antimicrobial activity. The enhanced antimicrobial activity of the complexes in relation to the ligand is due to chelation theory. Chelation results in an increase in the lipophilicity of the metal complexes, causing the concentration of complexes in the lipid membrane to increase and reducing microorganism multiplicity. It is hypothesized that the complexes’ antifungal effects result from either killing the bacteria or preventing their growth by obstructing their active sites [31,71].

Ahmed et al. synthesized the imine–iron complex **125** (Figure 1) in a 1:1 ratio with the ligand. The synthesized compounds were tested for their antimicrobial activity against the Gram-positive bacteria *S. aureus* and Gram-negative bacteria *E. coli*, as well as fungal strains *C. albicans* and *A. flavus*. All the selected microbes resisted the ligand except *E. coli*, against which it showed an inhibition zone of 9 mm, a value higher than that of the antibacterial drug amikacin (6 mm). Complex **125** showed the same zone of inhibition (10 mm) as the standard drug amikacin against *S. aureus* and enhanced activity against *E. coli* (10 mm) compared to the same standard drug. Several factors could be responsible for the remarkable antibacterial activity of the complex, including interference with the creation of the cell wall, harm because of which the permeability of the cell may be changed, or disorganization of the lipoprotein, resulting in cell death. Also, different cellular enzymes, essential in the metabolic pathways of microbes, could be deactivated. Another factor could be the formation of a hydrogen bond between the azomethine group and the active center of the cell’s components, interfering with proper cell function [72].

A mononuclear chelate of iron(III) was synthesized by Mohamed et al. by condensing a new tridentate Schiff base ligand (**128**) with iron chloride (FeCl_3_·H_2_O) in a 1:1 ratio. The complex formed (**129**) had an octahedral geometry. The in vitro antimicrobial potency of the synthesized complex (**129**) was evaluated against Gram-negative bacteria *E. coli* and Gram-positive bacteria *S. aureus* and fungal strains *C. albicans* and *A. flavus.* Complex **129** showed a broad zone of inhibition (14 mm/mg sample) against *A. flavus*, whereas the free Schiff base ligand demonstrated zero activity. This activity was much higher than that of ketoconazole (8 mm/mg sample), the selected standard antifungal agent. The enhanced microbial activity of the complex can be attributed to the increased lipophilicity of the metal complex upon coordination with the free ligand. This ensures the easy movement of the metal chelate into the fungal cell membrane, inhibiting microbial growth or distorting its active site [73,74]. For the other microbial strains, the Schiff base ligand showed activity similar to its free ligand (Table 2). The reason for the reduced activity of the Schiff base against *E. coli*, *S. aureus*, and *C. albicans* was not stated [75].

Hidayati et al. synthesized an N-(2-hydroxybenzylidene) chitosan Schiff base and its iron(II) complex and evaluated them for their antibacterial potency. Chitosan (poly-β-(1→4)-glucosamine) is a very abundant non-toxic natural biopolymer, and its metal complexes are known to exhibit very good biological activities. Hidayati et al. evaluated chitosan, the synthesized chitosan Schiff base ligand, and its imine complex for their ability to inhibit the growth of *E. coli* and *S. aureus* and found—at a concentration of 1000 ppm—the complex being most active against both bacterial strains (9.86 mm and 10.16 mm, respectively), followed by the chitosan Schiff base (9.50 mm and 9.33 mm, respectively) and lastly the chitosan itself (8.75 mm and 9.25 mm, respectively). The observed improvement in the antibacterial activity of the chitosan Schiff base–iron complex can be explained by chelation, which enhanced the lipophilic nature of the complex, ensuring its faster diffusion across bacterial cell membranes [9,76].

**Table 2:** Product, synthesis conditions, and in vitro antimicrobial activity of imine–iron complexes compared to the respective positive controls ^†^.

No.	Complex No.	Structures of Synthesized Complexes	Reaction Conditions	Antimicrobial Biological Activity							Ref.
1.	**51**	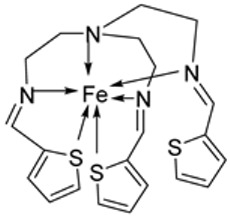	Stirring, 30 min Reflux, 7 h	Zone of inhibition, mm							[40]
					*S. aureus*	*E. coli*	*P. aeruginosa*	*B. cereus*			
				**50**	11	10	11	12			
				**51**	14	14	14	29			
				Tetracycline	9	10	12	11			
2.	**55**	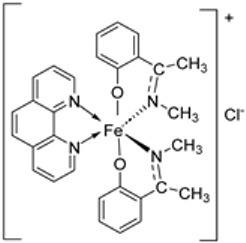	Stirring, 1–2 h Reflux, 2–11 h	Zone of inhibition, mm							[42]
						*E. coli*					
				**54**		23					
				**55**		29					
				Amoxicillin		41					
				Chloramphenicol		39					
3.	**34**	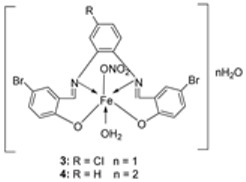	Reflux, 2 h	Minimum Inhibitory Concentration (MIC)/µg/mL							[21]
				Bacteria				Fungi			
					*S. marcescence*	*E. coli*	*M. luteus*	*G. candidum*	*A. flavus*	*F. oxysporum*	
				**1**	7.25	7.25	6.25	6.75	8.00	7.50	
				**2**	5.50	6.25	4.75	5.25	6.75	6.25	
				**3**	3.75	4.25	3.00	4.00	4.50	4.25	
				**4**	3.25	3.50	2.50	3.00	3.75	3.50	
4.	**59**	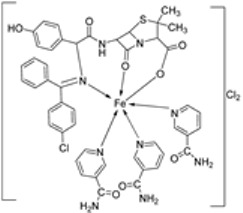	Stirring Reflux, 1 h	Minimum Inhibitory Concentration (MIC)/µg/mL							[47]
					*E. coli*	*Pseudomonas*		*S. aureus*		*Bacillus*	
				**58**	2.5	8		15		17	
				**59**	25	R		R		** R	
5.	**63** **64**	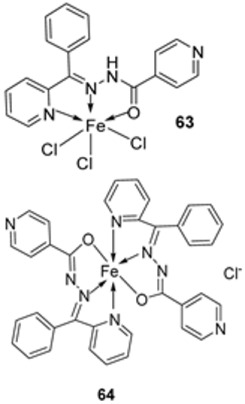	Stirring, 1 h Reflux, 9 h	Zone of inhibition, mm							[48]
						*Bacillus subtilis*				*E. coli*	
				**62**		11				15	
				**63**		12				11	
				**64**		14				18	
				Amoxicillin		16				20	
6.	**67**	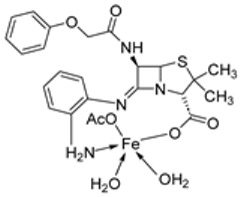	Reflux, 5 h	Zone of inhibition, mm							[65]
					*S. v*	*E. sp*	*S. a*	*E. f*		MRSA	
				**65**	15	24	16	17		R **	
				**67**	20	30	25	22		15	
				Standard	19	36	45	36		R	
7.	**68**	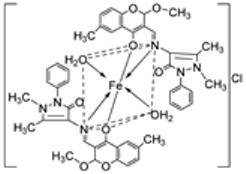	Reflux, 4 h	Minimum Inhibitory Concentration (MIC)/µg/mL							[10]
					*C. albicans*	*C. neoformans*	*S. aureus*	*B. cereus*		*E. coli*	
				**68**	0.0156	0.0078	0.0625	0.0312		0.0625	
				Nystatin	0.032	0.032					
				Miconazole	0.016	0.0162					
				Furacillinum							
				Ciprofloxacin			0.0046	0.0046		0.0046	
				Amikacin			0.001	0.0003		0.008	
8.	**69**	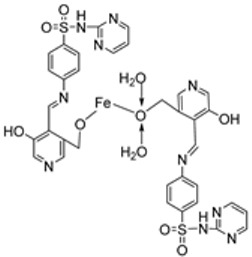	Reflux, 2 h	Zone of inhibition, mm							[51]
						*E. coli*	*E. aerogenes*			*C. butyrium*	
				Ligand		14	12			12	
				**69**		12	10			9	
				Standard		11	7			9	
9.	**73**	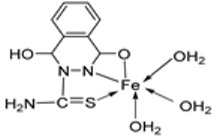	Stir and reflux, 1 h	Zone of inhibition, mm							[52]
						*S. pneumonia*		*S. aureus*			
				**72**		7–10		1–3			
				**73**		7–10		7–10			
10.	**76**	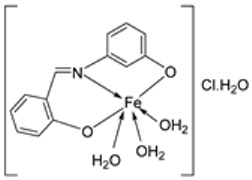	Stir and reflux, 2 h	Zone of inhibition, mm							[4]
					*S. aureus*	*E. coli*	*P. aeruginosa*	*C. albicans*		*A. fumigatus*	
				**75**	15	14	16	13		15	
				**76**	16	14	15	16		18	
				Ampicillin	23		16				
				Gentamycin							
				Amphotericin		19		25		23	
11.	**79**	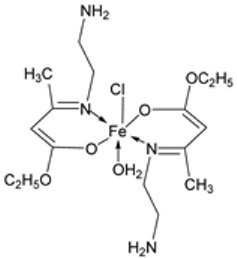	Reflux, 4 h	Zone of inhibition, mm							[53]
						*S. aureus*		*P. aeruginosa*			
				**78**		8		6			
				**79**		14		11			
				Ampicillin		14					
				Choloramphenicol				8			
12.	**82**	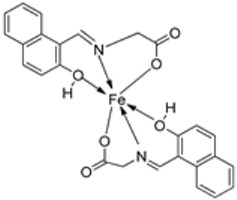	Stir (overnight)	Minimum Inhibitory Concentration (MIC)/µg/mL							[2]
					*E. coli*	*S. aureus*		*C. albicans*		*A. niger*	
				**82**	10	10		10		10	
				Gentamicin	10	10					
				Fluconazole				20		20	
13.	**83**	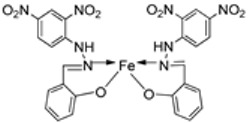	Reflux and stirring, 3 h	Zone of inhibition, mm							[54]
					*E. coli*	*P. aeruginosa*	*S. aureus*	*C. albicans*		*F. solani*	
				Ligand	R	R	12	^**^ R		^**^ R	
				**83**	14	8	12	7		12	
14.	**87**	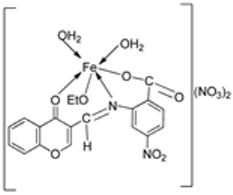	Stirring, 6 h	Minimum Inhibitory Concentration (MIC)/µg/mL							[3]
					*E. coli*	*C. albicans*	*P. vulgaris*	*K. pneumonia*		*S. aureus*	
				**86**	12.5	4	>50	1		>50	
				**87**	˃50	8	>50	>50		>50	
				Doxymycin Fluconazole	2		2	4		4	
						2					
15.	**88** **89**	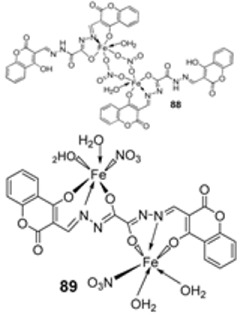	Stir, 30 min Reflux, 6 h	Zone of inhibition, mm							[55]
						*S. aureus*	*P. phaseolicol*			*F. oxysporium*	
				Ligand		22 ± 0.2	13 ± 0.1			17 ± 0.2	
				**88**		37 ± 0.4	26 ± 0.1			31 ± 0.2	
				**89**		32 ± 0.2	23 ± 0.1			30 ± 0.2	
				Cephalothin		42					
				Chloramphenicol			36				
				Cycloheximide						40	
16.	**96** **97**	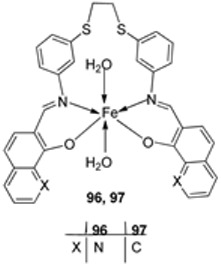	Reflux and stirring, 4–5 h	Zone of inhibition, mm							[57]
					*S. epidermidis*	*E. faecalis*	*S. aureus*	*P. mirabilis*		*C. albicans*	
				**94**	5	9	7	** R		** R	
				**95**	6	8	9	** R		** R	
				**96**	14	15	12	8		** R	
				**97**	12	8	7	22		** R	
				Amoxicillin	28	26	27	44			
17.	**100** **101**	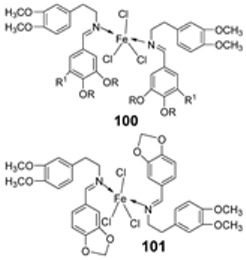	Reflux and stirring, 50 min	Zone of inhibition, mm							[59]
					*E. coli*	*P. aeruginosa*	*C. albicans*	*S. aureus*		*C. glabrata*	
				**98**	11	15	15	19		11	
				**99**	12	12	12	18		12	
				**100**	22	19	20	16		<10	
				**101**	20	16	13	20		12	
				Tetracycline	25	20		23			
				Nystatin			19			16	
18.	**105**	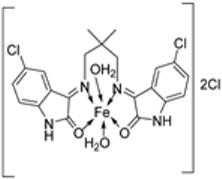	Reflux, 15 h	Zone of inhibition, mm							[60]
					*E. coli*	*S. epidermidis*	*A. niger*	*A. flavus*		*C. lunata*	
				**104**	** R	6	11	9		10	
				**105**	15	15	16	14		15	
19.	**34**	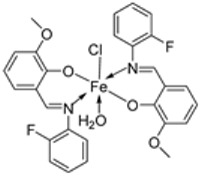	Reflux, 8–9 h	Zone of inhibition, mm							[30]
					*Bacillus*	*Staphylococcus*	*E. coli*	*S. rolfsii*		*M. phaseolina*	
				**33**	1	1	1	2		8	
				**34**	4	3	4	6		14	
				Streptomycin	9	11	5				
				Mancozeb				18		24	
20.	**109**	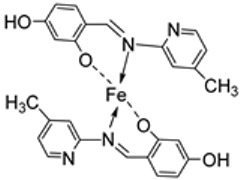	Reflux, 4–5 h	Zone of inhibition, mm							[61]
					*S. aureus*	*E. coli*	*A. niger*	*C. albicans*		*F. moniliforme*	
				**109**	3.02	** R	15.80	7.44		** R	
				Chloramphenicol	15.11	25.44		23.23			
				Amphotericin			15.78			12.58	
21.	**110**	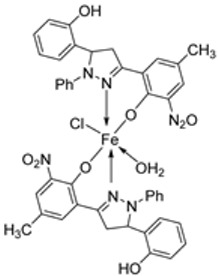	Reflux, 15–16 h	Zone of inhibition, mm							[62]
					*S. pyrogenes*	*E. coli*		*S. typhi*			
				**110**	25	16		19			
22.	**113**	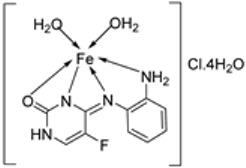	Reflux, 6 h	Zone of inhibition, mm (concentration, mg/mL)							[64]
					*B. subtilis*	*B. megaterium*	*P. aeroginosa*	*K. pneumonia*		*E. aerogenes*	
				**111**	40±0.47(0.2)	34±0.81(0.2)	42±1.24(1)	36±0.47(0.2)		45 ± 0.00	
				**112**	30 ± 0.81(0.2)	22±0.81(0.5)	33±0.81(0.2)	** R		28 ± 0.00	
				**113**	21 ± 0.00(0.2)	** R	36±1.24(1)	** R		** R	
				Erythromycin	20 ± 0.00	25±0.47	19±0.47	19±0.00		27 ± 1.24	
23.	**120** **121** **122**	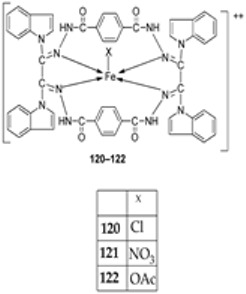	Reflux, 8 h	Zone of inhibition, mm							[66]
					*S. aureus*	*P. aureginosa*	*E. coli*	*S. typhii*	*Aspergillus* sp.	*Penicillium* sp.	
				**119**	36	08	10	10	48	29	
				**120**	30	36	41	42	68	61	
				**121**	24	25	22	28	51	54	
				**122**	62	65	33	35	80	66	
				Imipenem	100	100	100	100	57	65	
				Miconazole							
24.	**123**	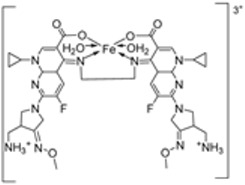	Reflux, 3 h	Zone of inhibition, mm							[35]
					*X. campestris*	*B. megaterium*	*C. michiganensis*	*M. fructicola*		*P. digitatum*	
				Ligand	30	28	32	36.0 ± 3.1		28.0±3.5	
				**123**	26	19	20	62.5 ± 6.2		62.5 ± 8.2	
				Tetracycline	34	28	30				
				Azoxystrobin				45.3 ± 2.1		58.1 ± 1.2	
25.	**124**	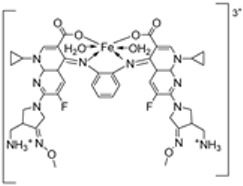	Reflux, 3 h	Zone of inhibition, mm (concentration, µg/mL)							[69]
					*E. coli*	*B. cereus*	*P. fluorescens*	*B. cinerea*		*A. flavus*	
				Ligand	20	12	11	0.0 ± 0.0		0.00 ±0.0	
				**124**	12	12	18	6.7 ± 2.3		6.7±2.6	
				Tetracycline	14	10	8				
				Cycloheximide				42.2±2.6		9.7±3.0	
26.	**30**	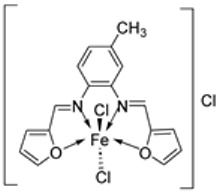	Stir and reflux, 4 h	Zone of inhibition, mm							[29]
					*S. aureus*	*B. subtilis*	*E. coli*	*C. albicans*			
				Ligand	19	25	24	25			
				**30**	17	16	19	15			
				Gentamycin	24	26	30				
				Ketoconazole				20			
27.	**125**	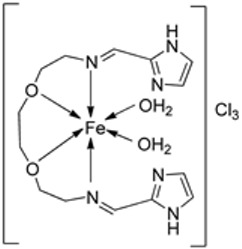	Stirring and reflux, 1 h	Zone of inhibition, mm							[72]
						*S. aureus*	*E. coli*				
				Ligand		0.00	9				
				**125**		10	10				
				Amikacin		10	6				
28.	**38** **39** **40**	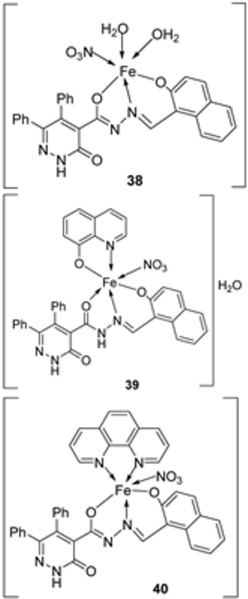	Stirring, 2 h Reflux, 12–15 h	Zone of inhibition, mm							[31]
						*S. typhimurium*		*C. albicans*			
				Ligand		** R		8			
				**38**		** R		14			
				**39**		15		22			
				**40**		** R		R			
				Cephalothin		36					
				Cycloheximide				35			
29.	**129**	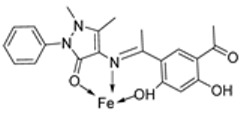	Stirring and reflux, 1 h		Zone of inhibition, mm/mg						[75]
					*E. coli*	*S. aureus*	*C. albicans*	*A. flavus*			
				**128**	14	12	10	0			
				**129**	13	11	12	14			
				Amikacin	6	10	-	-			
				Ketoconazole	-	-	9	8			

^†^ Values written as these have been reported in the literature. ** R = 0 = Resistant.

### 2.3. Imine–Iron Complexes as Antioxidants

Oxidative metabolism is one of the crucial factors for cell survival. Free radicals and other reactive oxygen species (ROS) are produced because of this reliance, which leads to oxidative alterations. When too many free radicals are produced, the ROS concentration becomes above average, which can overwhelm protective enzymes and have detrimental and fatal effects on cells by oxidizing membrane lipids, cellular proteins, DNA, and enzymes, which stops cellular respiration [18]. The way to counter the reaction of these free radicals is to introduce an antioxidant, which can be experimentally carried out using assays such as DPPH [77]. Compounds of metal chelates like iron–imine complexes offer advantages over conventional synthetic antioxidants because of the different geometry, oxidation states, and coordination numbers of metal chelates that support and promote the redox processes linked to antioxidant action. In its high oxidation state, the metal ion’s ability to extract electrons influences the antioxidant activity of the resultant complex by moving the ligand’s electron density to the metal center, where it functions as a modulator agent. When the metal is in its low oxidation state, it behaves in the opposite way. These actions significantly alter the ligand’s electrical charge distribution, facilitating the loss of electrons and raising the complex’s capacity to scavenge radicals. A metal ion like iron (Fe), which is found in many biologically privileged structures and essential to an organism’s ability to function, is useful in constructing novel chelate antioxidants because it lowers toxicity [78].

Turan et al. synthesized an imine ligand and its octahedral imine–iron(II) complex **126** (Figure 1) and evaluated their in vitro antioxidant activity using 2,2-diphenyl-1-picryl-hydrazyl-hydrate (DPPH) radical scavenging, 2,2’-azino-*bis*(3-ethylbenzothiazoline-6-sulfonic acid) (ABTS) cation radical scavenging, and the ferric reducing ability of plasma (FRAP). In the ABTS assay, a compound’s antioxidant ability is measured based on the reduction of ABTS•+ cation radicals [79]. Complex **126** (0.6) demonstrated weak ABTS•+ radical scavenging activity, while the parent ligand exhibited no discernible ABTS•+ radical scavenging activity. The molecule’s structure and single electron transfer potential influence the results. The complex showed a more enhanced DPPH radical scavenging ability (1.25) than the ligand (1.35) itself, but this activity was moderate when compared with the standard drugs (0.10–0.31). The FRAP method measures a compound’s ability to cause the reduction of ferric ions (Fe^3+^) to ferrous ions (Fe^2+^). The ligand (0.5) showed an antioxidant ability similar to that of **126** (0.4) in this assay (Table 3). The antioxidant potency of a series of compounds has been studied for the potential that they can be influenced by the aromatic ring and the number of hydroxyl groups present in a compound [80,81].

El-Lateef et al. explored imine–iron complexes **3** and **4** (Scheme 1) for their antioxidant activity using the DPPH assay. The results revealed that the free ligand and its metal complexes have better antioxidant activity than the standard antioxidant agent vitamin C (55 μg/mL). The complexes showed enhanced activity compared to the free ligands (**1** = 45µg/mL, **2** = 32 µg/mL), with **3** possessing the highest DPPH free radical scavenging ability with an IC_50_ value of 22μg/mL (Table 3). The results indicate that the complexes had greater antioxidant effects against the DPPH free radical than standard vitamin C and can be considered antioxidant drug candidates. This study did not state the reason for the exceptional activities of the ligands and complexes [21].

Naureen et al. explored the antioxidant activity of **100** and **101** (Scheme 19) using the DPPH assay. The free imine ligands **98** and **99** demonstrated better antioxidant activity (1.23 and 1.02 μg/mL, respectively) than their iron complexes, **100** (1.70 μg/mL) and **101** (1.41 μg/mL). The free ligand **99** showed better antioxidant activity (1.02 μg/mL) than the standard vitamin C (1.14 μg/mL). Generally, both the free ligand and iron complexes exhibited good free radical scavenging abilities (Table 3). The modes of action of the ligands and their complexes were not outlined [59].

**Scheme 24:**
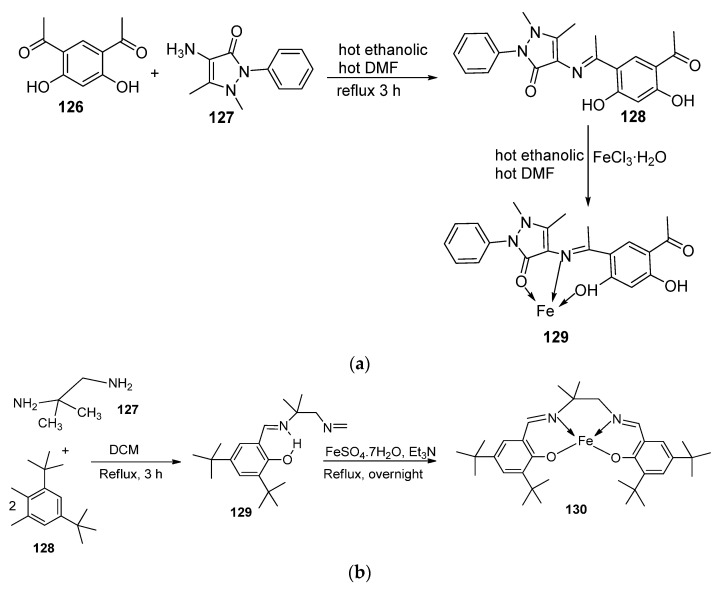
(**a**) Synthesis of complex **129**. (**b**) Synthesis of complex **130**.

The tetradentate Schiff base **129** was synthesized along with its Fe complex **130** (Scheme 24) by Said et al. An in vitro antioxidant activity was determined using the DPPH radical scavenging, ferric thiocyanate (FTC), hydroxyl radical scavenging activity (HRSA), and hydrogen peroxide scavenging activity methods. Complex **129** demonstrated a better free radical scavenging ability than the synthesized complex **130** in the DPPH radical scavenging, FTC, and HRSA methods with IC_50_ values of 53.55, 48.81, and 63.43, respectively, whereas those of **130** were 44.65, 9.47, and 30.29. The complex showed moderate activity compared to the standard Trolox and BHA in the DPPH radical scavenging, FTC, and HRSA methods (Table 3). It, however, demonstrated a better ability (93.74 μg/mL) to remove H_2_O_2_ from the reaction mixture than **129** (92.52 μg/mL) and the standards Trolox (91.80 μg/mL) and BHA (92.97 μg/mL) when the hydrogen peroxide scavenging activity method was employed (Table 3). Due to the presence of the hydroxyl group on the ligand, its antioxidant activity was expected to be higher than that observed in this study, and this may be due to the steric hindrance or the presence of bulky donating groups (or both), making it challenging for the ligand to supply the hydrogen atom (H) to the DPPH radical [82].

**Scheme 25:**
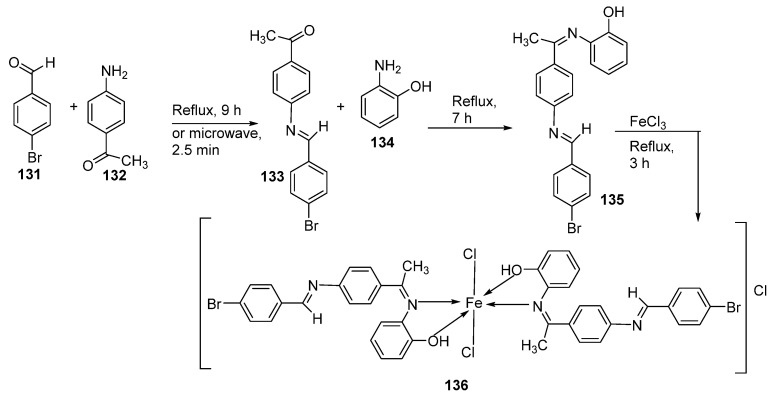
Synthesis of complex **136**.

Hayder et al. synthesized the new imine ligand **135** and its octahedral imine–iron complex **136** (Scheme 25). The antioxidant activity of **135** and **136** was evaluated using the DPPH radical scavenging activity method. The iron complex showed an enhanced ability to scavenge DPPHꞏ radicals (49% scavenging) than the free ligand (24% scavenging). Compared to the standard ascorbic acid (82% scavenging), the complex showed a moderate ability to scavenge the free radicals in the reaction mixture (Table 3) [16].

Elshafie et al. evaluated complex **124** (Figure 1) for its in vitro antioxidant activity. The free imine ligand and complex **124** both showed a high antioxidant activity (164.6%), with the iron complex being slightly higher than the ligand (169.7%). Complex **124** can donate hydrogen to scavenge the free radicals, hence reducing the oxidation process (Table 3) [69].

Borase et al. conducted an antioxidant assay on the metal complex **109** (Scheme 21) to determine its free radical scavenging ability, and it proved to have moderate antioxidant activity (1615.22 μg/mL) (Table 3). The results of the antioxidant activity of ligands were not given, and subsequent comparisons could not be made [61].

Savcı et al. investigated **111**, imine ligand **112**, and its imine–iron complex **113** (Scheme 22) for their antioxidant activity using DPPH radical scavenging, total antioxidant activity, FRAP, and CUPRAC activity. The results obtained revealed that the iron complex **113** (0.7) had a high ability to remove DPPH from the reaction mixture when compared to **111** (1.9), **112** (0.8), and the standard BHT (1.1). For the total antioxidant activity assay, both **112** (0.62)) and **113** (0.61) showed a similar potential to the standard BHA (0.60) in eliminating lipid peroxide from the reaction mixture and an enhanced potential compared to the standard BHT (0.40). In the FRAP assay, **111** (0.06) showed the lowest reduction capacity in reducing the Fe^3+^ ions, followed by the standard BHT (0.08), complex (0.11), and BHA (0.2), and the ligand **112** (0.38) showed the highest activity. Finally, the CUPRAC method confirmed the results of the other assays, with **111** indicating the lowest antioxidant activity (Table 3). Most of the inhibitor’s antioxidant effect comes from its ability to donate one electron or hydrogen to the radical centers formed in biological systems, thus neutralizing them. The inhibitor’s structure and characteristics are critical factors in demonstrating activity [49]. Potential sites for biochemically active substances connected to the balance of molecular proton transfer and hydrogen bonds can be found in the Schiff bases. The biological activity of the Schiff base [83] is typically increased by complexes formed with transition metals. Hence, the good antioxidant activity of both the ligand and complex was achieved in this study [84].

**Table 3:** Products, synthesis conditions, and antioxidant activity of selected imine–iron complexes using DPPH, H_2_O_2_SA (hydrogen peroxide scavenging activity assay), %RSA (radical scavenging activity), and total antioxidant assay (TAC) ^†^.

Entry No.	Complex No.	Structures	Reaction Condition	Antioxidant Activity (IC_50_/µg/mL)						Ref.
1.	**3** **4**	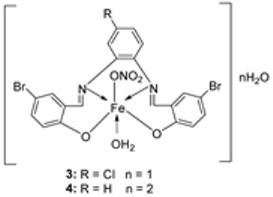	Reflux (2 h)		DPPH					[21]
				**1**	45					
				**3**	22					
				**2**	53					
				**4**	32					
				Vit C	55					
2.	**100** **101**	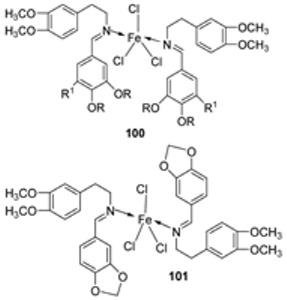	Reflux and stirring (50 min)		DPPH					[59]
				**98**	1.23					
				**100**	1.70					
				**99**	1.02					
				**101**	1.41					
				Vit C	1.14					
3.	**126**	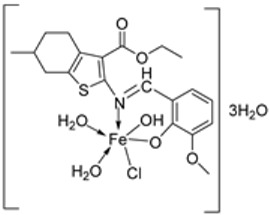	Reflux (10 min) and stirring (24 h)		ABTS (734 nm)	DPPH (517 nm)		FRAP (700 nm)		[81]
				Ligand	1.90	1.35		0.50		
				**126**	0.60	1.25		0.40		
				Ascorbic acid	0.00	0.10		2.10		
				BHA	0.00	0.18		2.90		
				BHT	0.00	0.31		2.30		
4.	**130**	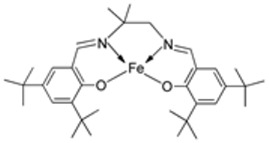	Reflux and stir (overnight)		DPPH	H_2_O_2_ SA	FTC		HRSA	[82]
				**129**	53.55 ± 2.95	92.52 ± 0.07	48.81 ± 5.04		63.43 ± 5.66	
				**130**	44.65 ± 1.10	93.74 ± 0.43	9.47 ± 2.191		30.29 ± 0.81	
				Trolox	85.42 ± 0.04	91.80 ± 1.77	90.45 ± 6.70		57.72 ± 1.62	
				BHA	75.69 ± 0.11	92.97 ± 0.98	50.57 ± 5.42		10.00 ± 3.64	
5.	**136**	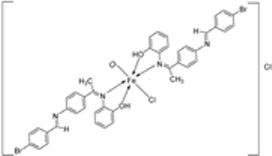	Reflux (3 h)		DPPH (% scavenging)					[16]
				**135**	24					
				**136**	49					
				Ascorbic acid	82					
6.	**124**	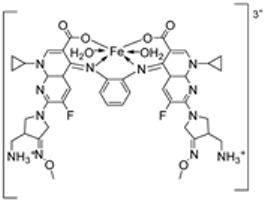	Reflux (3 h)		%RSA					[69]
				Ligand	169.7					
				**124**	164.6					
7.	**109**	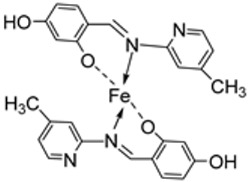	Reflux (4–5 h)		DPPH					[61]
				**109**	1615.22					
8.	**113**	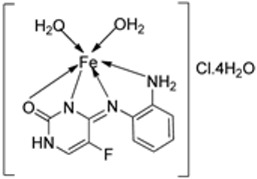	Reflux (6 h)		DPPH	Total antioxidant	FRAP		CUPRAC	[64]
				**111**	1.9	0.64	0.06		0.30	
				**112**	0.8	0.62	0.38		3.50	
				**113**	0.70	0.61	0.11		1.20	
				BHT	0.60	0.60	0.08		3.20	
				BHA	1.20	0.44	0.20		3.10	

^†^ Values are written as reported in the literature. DPPH: 2,2-diphenylpicrylhydrazyl, FTC: ferric thiocyanate, FRAP: Ferric Reducing Antioxidant Power, CUPRAC: CUPric Reducing Antioxidant Capacity, BHA: beta hydroxy acid, BHT: butylated hydroxytoluene, HRSA: hydroxyl radical scavenging activity.

### 2.4. Other Pharmacological Activities of the Imine–Iron Complexes

Iron–imine complexes have been found to possess anti-inflammatory properties, and their mechanism of action has been studied extensively. One of the primary mechanisms by which these complexes exhibit anti-inflammatory activity is inhibiting the production of pro-inflammatory cytokines such as TNF-α, IL-1β, and IL-6. These cytokines play a crucial role in the inflammatory response by recruiting immune cells to the site of inflammation and activating them. By inhibiting the production of these cytokines, iron–imine complexes can reduce the activity of immune cells and thus reduce inflammation. Additionally, iron–imine complexes have been found to inhibit the activity of cyclooxygenase-2 (COX-2), an enzyme that plays a key role in producing prostaglandins. Prostaglandins are lipid mediators that are involved in the inflammatory response, and their production is increased during inflammation. By inhibiting COX-2 activity, iron–imine complexes can reduce the production of prostaglandins and thus reduce inflammation. Iron–imine complexes have also been found to inhibit the activity of NF-κβ, a transcription factor that plays a crucial role in regulating immune and inflammatory responses. NF-κβ is activated in response to various stimuli, including pro-inflammatory cytokines, and its activation results in the transcription of genes involved in the inflammatory response. By inhibiting NF-κβ activity, iron–imine complexes can reduce the expression of inflammatory genes and thus reduce inflammation.

Kumar et al. screened imine–iron complexes **120**, **121**, and **122** (Scheme 23) for their in vivo anti-inflammatory activity using albino rats. All the complexes showed anti-inflammatory activity higher than that of the standard drug phenyl butazone (18.2% anti-inflammatory activity), with **122** (31.1%) exhibiting the highest activity at the same concentration of 25 mg/kg. Complex **121** showed the least anti-inflammatory activity (27.2%). Compared to the complexes, the ligand (9.0%) showed very low anti-inflammatory activity (Table 4). Complex **122** can be explored further as an anti-inflammatory drug candidate. The increased anti-inflammatory activity of the complex in relation to the ligand can be explained by the chelation theory, which describes the increase in polarity and the lipophilic nature of the complex due to chelation and how this causes it to efficiently cross the lipid layer, affecting the desired anti-inflammatory action [66].

Imine–iron complexes have shown a few other medicinal activities. Ahmed et al. screened complex **125** (Figure 1) on coronavirus (SARS-CoV-2) using molecular docking. The molecular docking studies investigated the interaction that exists between the complex and the crystal structure of the virus’s (SARS-CoV-2) main protease with unliganded active site (2019-nCoV, coronavirus disease 2019, or COVID-19) (PDB ID: 6Y84) proteins. The imine–iron complex **125** (Figure 1) had low energy, (−8.5 kcal/mol), which means it has a strong binding affinity and can inhibit the biochemical processes of the proteins, inhibiting viral capability (Table 4) [72].

**Scheme 26:**
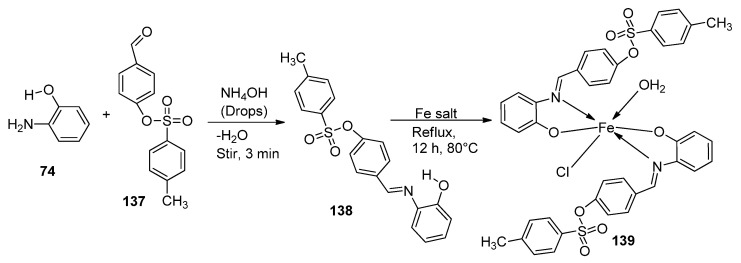
Synthesis of complex **139**.

Elkanzi et al. synthesized imine–iron complex **139** (Scheme 26) and screened its in vitro anti-inflammatory activity using the anti-denaturation method of egg albumin. Heat applied to the egg denatures the egg albumin, and the denatured protein produces certain antigens. These antigens are linked to type-III hypersensitivity reactions, which cause several diseases. The anti-inflammatory assay analyses test an agent’s ability to limit the denaturation process. The result obtained from this study showed that **139** exhibited a moderate percentage of inhibition (0.70) as compared to the ligand (0.13) and standard anti-inflammatory drug ibuprofen (2.9) at a concentration of 100 µg/mL (Table 4). The inverse relationship between the dipole moment of the complex and its activity explains the reduced activity of the complex. The dipole moment of the complex (10.11) is higher than that of the ligand (5.91), and this increases the polarity and decreases the lipophilic nature of the complex, lowering its efficiency to passing through the lipid layer, hence making it less efficient as an anti-inflammatory agent [83,84].

**Table 4:** Products, synthesis conditions, and biological activities of selected imine–iron complexes ^†^.

Entry No.	Complex No.	Structures	Reaction Conditions	Biological Activities		Ref
1.	**120** **121** **122**	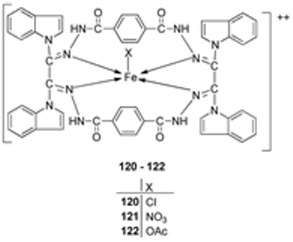	Reflux, 8 h		Anti-inflammatory activity (%)	[66]
				**119**	9.00	
				**120**	28.50	
				**121**	27.20	
				**122**	31.10	
				Phenyl butazone	18.20	
2.	**125**	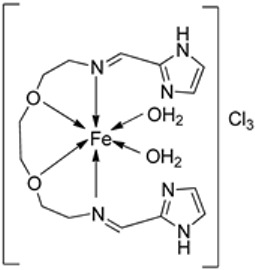	Stirring and reflux, 1 h		Binding energy (kcal/mol)	[72]
				Ligand	−2.1	
				**125**	−8.5	
3.	**139**	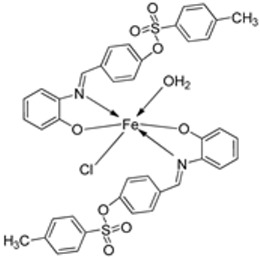	Reflux, 12 h		% Inhibition of heat-induced denaturation of proteins	[84]
				**138**	0.13	
				**139**	0.70	
				Ibuprofen	2.9	

^†^ Values are written as reported in the literature.

## 3. Conclusions

While nitrogen is a vital macro-nutrient, iron is a significant micro-nutrient in the human body. Accordingly, the biocompatibility (possibility of being bioavailable) of iron–imine complexes in the human body is higher than that of other organometallic complexes. This article discusses the recent development of organo-iron compounds as medicinally privileged compounds. As discussed herein, some of the iron–imine complexes demonstrated good-to-excellent pharmacological activity in several dreadful diseases like different types of cancers and microbial, oxidation, and inflammation-related diseases. The observed anticancer activity of iron–imine complexes is believed to be due to their tailored delivery and different mechanisms of action, which include altering iron metabolism, producing reactive oxygen species (ROS), and blocking key enzymes. As antimicrobial agents, they outperform many conventional antimicrobial agents due to novel mechanisms of action, broad-spectrum activity, and biofilm disruption, which can be traced to the chelation process, the toxicity of metal ions against bacteria, and the improvement in the hydrophobicity and liposolubility of the molecules due to the presence of an azomethine linkage in the complex. The different geometry, oxidation states, and coordination numbers of metal chelates like iron complexes support and promote the redox processes linked to antioxidant action. One of the primary mechanisms by which these complexes exhibit anti-inflammatory activity is inhibiting the production of pro-inflammatory cytokines such as TNF-α, IL-1β, and IL-6. They also inhibit the activity of cyclooxygenase-2 (COX-2). This enzyme plays a key role in the production of prostaglandins (lipid mediators that are involved in the inflammatory response, and their production is increased during inflammation).

Iron (Fe), complex in many biological structures and essential to an organism’s ability to function, can be useful in constructing novel chelate drugs because it can possibly lower toxicity. The chemistry and biology of iron is still under investigation. Quite recently, a unique version of an iron-dependent non-apoptotic cell death procedure was reported [85,86]. Iron–imine complexes can be developed as a valuable probe for antimicrobial, antifungal, anti-inflammatory, and antioxidant drug development. Accordingly, iron–imine complexes can play a crucial role in future drug development research. In a nutshell, iron, the fourth most abundant element in the Earth’s crust, can form various non-toxic complexes with imines. Iron–imine complexes have demonstrated diverse medicinal activities, and some of these derivatives have shown promise in becoming commercial drugs in the future.

## Data Availability

All data are available online. No unpublished data have been used in this paper.
